# Small-molecule correlates of infection precede infection diagnosis in breast implant reconstruction patients

**DOI:** 10.1172/JCI192104

**Published:** 2025-12-23

**Authors:** John A. Wildenthal, Margaret A. Olsen, Hung D. Tran, John I. Robinson, Terence M. Myckatyn, David K. Warren, Keith E. Brandt, Marissa M. Tenenbaum, Joani M. Christensen, Thomas H. Tung, Justin M. Sacks, Rachel A. Anolik, Katelin B. Nickel, Hideji Fujiwara, Peter J. Mucha, Jeffrey P. Henderson

**Affiliations:** 1 Infectious Diseases Division, Department of Medicine and; 2 Division of Plastic and Reconstructive Surgery, Washington University in St. Louis School of Medicine, St. Louis, Missouri, USA.; 3 Department of Mathematics, Dartmouth College, Hanover, New Hampshire, USA.

**Keywords:** Infectious disease, Inflammation, Microbiology, Bacterial infections, Metabolomics, Surgery

## Abstract

**BACKGROUND:**

Infection is an important complication of implanted devices and prosthetics. Identifying infections sufficiently early to salvage implants and avoid reconstructive failure is a persistent medical challenge.

**METHODS:**

Two cohorts of women 21 years and older undergoing breast implant reconstruction were recruited. Seroma fluid (82 breasts, 70 patients) was collected upon implant removal for infectious or noninfectious causes. Postimplantation drain fluid (100 samples, 44 breasts, 32 patients) was collected at routine visits prior to implant removal. A liquid chromatography/mass spectrometry–based metabolomic approach was used to identify infection correlates.

**RESULTS:**

In seroma fluid specimens, infection was associated with a diverse set of small molecules, including acetylated polyamines, defensins, glucosyl-sphingosine, and several peptide-like features (all *P* < 0.001, diagnostic areas under the receiver operating curve 0.82–0.93). Notably, a subset of these markers were significantly elevated (*P* < 0.05) in postimplantation drain fluid before recorded infection symptoms and diagnosis. *Pseudomonas aeruginosa* and its specialized exometabolites in drain specimens were also associated with subsequent *P*. *aeruginosa* infections.

**CONCLUSION:**

Tissue fluid from infected patients has a distinctive metabolome reflecting human and bacterial physiologic processes that often precede clinical diagnoses. A diagnostic based on these findings has potential to improve patient outcomes through early recognition of infection.

**FUNDING:**

This work was supported by U54CK000609 from the CDC and by an unencumbered research gift from Sientra. Metabolomic approaches were supported by NIH grants R01 DK125860 and R01 DK111930.

## Abstract 2

Metabolomic profiling of tissue fluid from breast reconstruction patients revealed an infection-associated molecular signature that, in longitudinal samples, substantially pre-dated clinical infection diagnosis.

## Introduction

It is axiomatic among infectious disease physicians that antimicrobial therapy is most effective when delivered early in infection. This principle has guided practice and early recognition efforts for pneumonia, septic shock, necrotizing fasciitis, and breast implant–associated infections (1–8). An important barrier to early therapy, however, is diagnostic, such that infection therapies are generally not considered until definitive symptoms appear. For many infection types, it is unclear exactly when the infection process begins relative to the appearance of symptoms. This is particularly true for implant-associated infections, which can appear weeks to months after implant placement (7, 9–12). The events preceding infection in human populations are difficult to study, as relevant specimens from future infection sites are, understandably, difficult to obtain prior to the development of signs and symptoms.

Early recognition of infection is especially challenging in patients undergoing breast implant reconstruction, where infectious signs and symptoms such as erythema, warmth, and tenderness overlap with noninfectious postsurgical inflammation or sequelae from radiation therapy (7). The “gold standard” definition of breast surgical site infection by the CDC relies upon either purulent drainage, abscess, or a positive aseptically collected culture (13, 14). Aseptic cultures are often only obtained at the time of implant removal and after antibiotic administration, a diagnostic delay that leads to high (~25%) rates of false negative cultures (13–15). Clinical diagnosis by a surgeon or attending physician is also part of the CDC definition, though the physician criteria for infection vary widely by institution and study (13, 16, 17). Breast implant reconstruction infections can lead to numerous undesired outcomes, including unplanned operations, increased cost, worse cosmetic outcomes, delay of adjuvant cancer therapies, and patient-directed abandonment of reconstructive efforts (18–22). An accurate and rapid implant-associated infection diagnostic that is deployable before definitive clinical symptoms would have the potential to avoid substantial patient morbidity and cost.

Here, we seek a molecular signature of infection applicable to patient fluid specimens. Rather than using animal models of implant-associated infection, which are subject to species-specific immune responses (23–26), we opted to pursue discovery analyses using specimens from patient cohorts. For this study design, breast implant reconstruction offers several advantages over other implant-associated infections. Breast implant reconstruction has high rates of infection, varying from 3% to 20% (27, 28), compared with approximately 1% to 2% for orthopedic joint infections (10). Two-stage reconstruction, in which tissue expanders (TEs) are placed and then removed for subsequent definitive reconstruction (29), permits sampling and comparative analysis of both infected and uninfected implants. This is not an option for permanent hardware such as joint or spine implants, which are rarely removed or sampled in uninfected patients. Breast implant reconstruction also involves fluid drainage using surgical drain placement into the breast pocket after mastectomy for 1 to 4 weeks, when patients are at highest risk for infection (12, 30). Specimen access in this patient population thus offers the potential to discern molecular features of infection pathogenesis in implant-associated infections.

To discern a molecular signature useful for implant infection diagnosis, we elected to use mass spectrometry–based small-molecule profiling (metabolomic profiling). This approach identifies and quantifies metabolites, lipids, small peptides, hormones, and microbial exometabolites that may reflect inflammatory and infection-related processes (31–34). These metabolites may be discovered in an untargeted manner, without a priori knowledge of the molecule, which is valuable given the many pathophysiologic unknowns in implant-associated infections. Another key feature of this analytical approach is its ability to provide quantitative data on individual molecules, which may resolve finer distinctions in specimens representing early infection pathophysiology. Indeed, these mass spectrometry tools have a rich history in informing pharmacokinetic modeling (35). Numerous recent examples of specific host and bacterial small molecules in infections reinforce the potential of this approach (36–39).

In this study, we collected breast pocket seroma fluid from 82 breasts in 70 women at the time of implant removal and 100 longitudinal drain fluid specimens from 44 breasts in 32 patients before implant removal. High-resolution liquid chromatography–mass spectrometry (LC-MS) analyses revealed metabolites from several classes that differentiate infected from noninfected implants at the time of implant removal. We found that a subset of these markers distinguished breasts that subsequently proceeded to clinical infection in the longitudinal samples. Additional findings raise the possibility that these early infection indicators represent nascent, subclinical infections. In addition to identifying new molecular correlates of human bacterial infections, these results provide the basis for identifying infection pathophysiology at an early stage, when intervention could improve patient outcomes.

## Results

### Clinical cohort.

**Figure 1:**
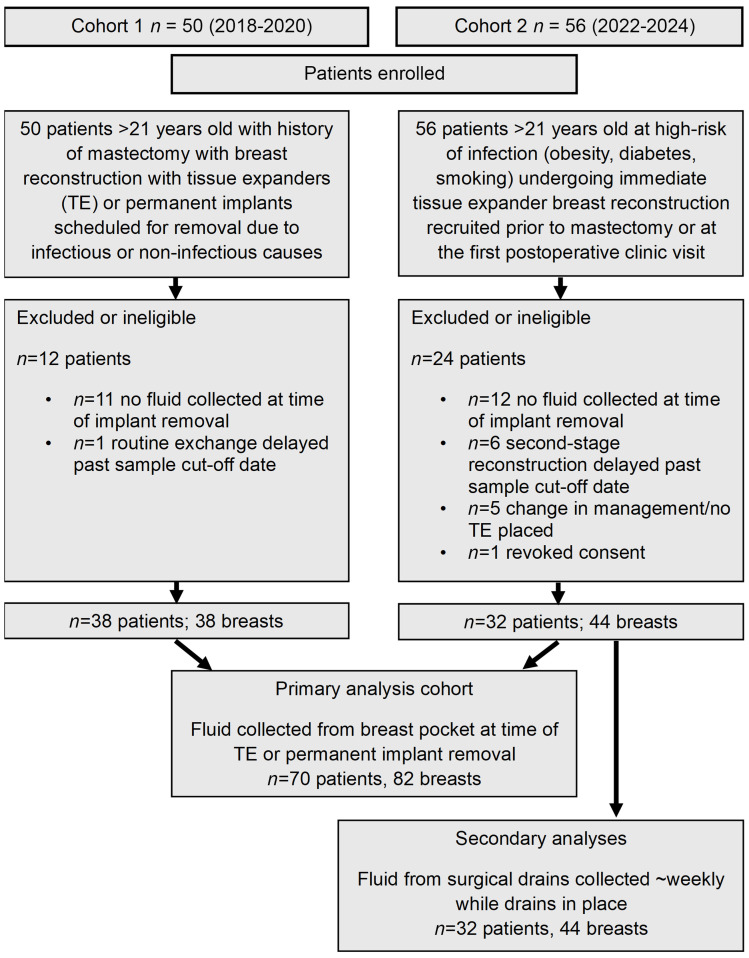
Recruitment strategy and sample collection overview.

We enrolled 106 women in 2 distinct clinical cohorts, termed cohorts 1 and 2 (Figure 1). Cohort 1 recruited 50 women from 2018 to 2020 at the time of removal of TEs or permanent breast implants. Since women were recruited at the time of explantation, the reason for implant removal (i.e., infection, noninfectious wound complication, desire to end further reconstruction, or removal at the time of second-stage reconstruction) was already known, and women with clinical implant infection diagnoses were prioritized for recruitment. Cohort 2 recruited 56 women from 2022 to 2023 at the time of mastectomy who were undergoing TE-based reconstruction and were at higher risk of infection (with 1 or more of the following risk factors: obesity, diabetes, or current/recurrent tobacco use). Because women were recruited at the time of mastectomy with implant placement for cohort 2, they were followed longitudinally until the time of TE removal or the sample cutoff date. For both cohorts, implants and breast pocket seroma fluid (if any) were collected by the surgeons in the operating room at the time of implant removal and transported to the laboratory for immediate microbiologic culture workup. Because the seroma fluid specimens were collected and processed identically in the laboratory for the 2 cohorts (see Methods), the metabolomic analyses using the remaining frozen seroma fluids were performed together and results from the 2 cohorts were combined. In cohort 2, additional longitudinal drain fluid samples were collected at routine postmastectomy clinic visits prior to drain removal. Patients were excluded from analysis (*n* = 36 total) if no seroma fluid was present for metabolomic analysis from the breast pocket at time of implant removal (*n* = 23), TE removal for second-stage reconstruction was delayed past the sample cutoff date (*n* = 7), no TE was placed due to a change in clinical management plans (*n* = 5), or due to revoked consent (*n* = 1).

Including both cohorts, implants and intraoperative seroma fluid were available from 82 breasts in 70 patients. In total, 36 specimens met our criteria for removal due to infectious causes (see Methods) and 46 specimens for noninfectious causes (34 planned removal for second-stage reconstruction, 10 for noninfectious complications, and 2 from patients with elective removal of the implant contralateral to an infected breast). These 82 intraoperative specimens served as our gold standard samples to assess fluid metabolomes for diagnostic markers, similar to prior studies on breast implant reconstruction infection (40–42). Patient-level demographic information and breast-specific clinical information for the final combined clinical cohort is shown in Table 1 and Table 2, respectively. In addition, across the 44 breasts (32 patients) in cohort 2, we collected 100 longitudinal specimens of fluid approximately weekly from surgical drains placed after mastectomy and immediate reconstruction prior to drain removal. Drain collections past the third clinic visit were excluded due to low numbers.

### Bacterial cultures of specimens at time of implant removal.

To identify the bacteria most likely to have caused implant infections in our patient population, we cultured seroma fluid samples from the breast pocket and swabbed the explanted implants/TEs for all 82 breasts in our study. All seroma fluids and implants from both cohorts were kept at room temperature until processing for microbial culture, with 79% cultured within 5 hours of specimen collection. A breast was considered culture positive if a bacterium grew from either implant swabs or from seroma fluid. Culture results for the combined cohorts are shown in Table 3. For each bacterial species, recovery in monomicrobial and polymicrobial culture results are shown in Supplemental Table 1 (supplemental material available online with this article; https://doi.org/10.1172/JCI192104DS1). For infected breasts (*n* = 36), gram-positive bacteria were the most commonly isolated organisms led by *Staphylococcus* species (*n* = 16, 44.4%). One or more gram-negative organisms were cultured from 13 (36.1%) infected breasts. The most commonly recovered gram-negative species was *Pseudomonas aeruginosa* (*n* = 8, 22.2%). No organisms were cultured from 4 infected breasts (11.1%). Most specimens with *Staphylococcus aureus* and *P*. *aeruginosa*, species with commonly assumed pathogenic roles, were recovered in monomicrobial cultures (Supplemental Table 1). For implants removed at the time of planned second-stage reconstruction in breasts without evidence of infection (*n* = 34), there was no growth from 20 breasts (58.8%). For implants without evidence of infection that grew 1 or more bacteria (*n* = 14, 41.2%), skin commensals such as coagulase-negative *Staphylococcus* (*n* = 6, 17.6%) and *Cutibacterium* species (*n* = 9, 26.5%) were the most common isolates. For implant/TE removal due to noninfectious wound complications (e.g., tissue necrosis and/or dehiscence, *n* = 10), culture results were more variable and included gram-negative bacteria such as *Proteus mirabilis* (*n* = 1, 10%), *P*. *aeruginosa* (*n* = 2, 20%), and *Enterobacter* species (*n* = 1, 10%), with no growth in culture of specimens from 5 breasts (50%). Overall, the bacteriology of our clinical cohort was diverse, including both gram-positive and -negative species.

### Metabolomic dataset overview.

**Figure 2:**
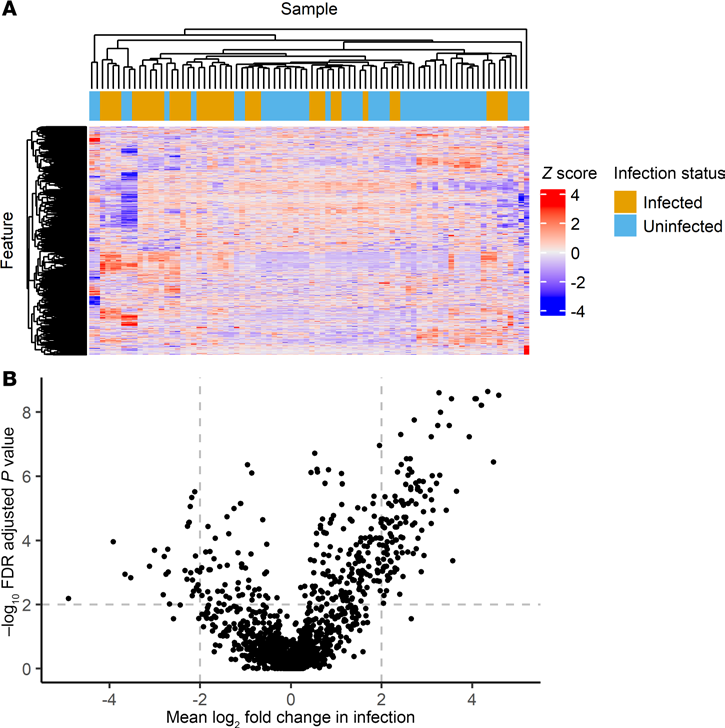
Untargeted metabolomic analysis of seroma fluid specimens collected at implant removal. (**A**) Heatmap of 2,540 features considered for untargeted metabolomic discovery. Color denotes centered and scaled (*z* score) feature intensity in each sample. (**B**) Volcano plot of features considered for untargeted metabolomic discovery. The *x* axis is the mean log_2_ fold change between specimens collected from infected breasts when compared with specimens collected from breasts with implants removed for noninfectious reasons. The *y* axis is the adjusted –log_10_ Wilcoxon’s rank sum *P* value adjusted for all 2,540 features using the BH method.

To identify metabolomic markers that differentiate implant infection status, we first used untargeted LC-MS to detect and quantify small molecules in intraoperative seroma fluid stored frozen at –80°C from the combined cohorts. Preprocessing the resulting profiles to minimize duplicate signals and restricting to high-quality features, as described in Methods, yielded 2,540 features for further analysis. Initial hierarchical clustering and heatmap-based analysis did not clearly distinguish infection status (Figure 2A), suggesting that infection was not the predominant driver of metabolomic variation in these samples. To discern infection-associated metabolite features, we used volcano plot analysis (Figure 2B), which revealed 115 features significantly increased at least 4-fold with an FDR-adjusted (Benjamini-Hochberg [BH] method) *P* (Wilcoxon’s rank sum) < 0.01 in samples from infected breasts. Conversely, 30 features were decreased by at least 4-fold with an FDR-adjusted *P* < 0.01. To identify features that are robust to variations in physiological dilution, we evaluated pairwise log ratios of feature abundances using differential compositional variation (DCV) scores in the SelEnergyPerm (43) compositional data analysis pipeline. The impact of potential outlier specimens on feature selection was minimized by calculating DCV scores on 25 random 75% subsamples of our dataset and retaining features ranked within the top 20 DCV scores in at least 50% of samplings. This procedure identified 16 features, all of which were increased in infection (Table 4).

### Positive molecular correlates of infection.

**Figure 3:**
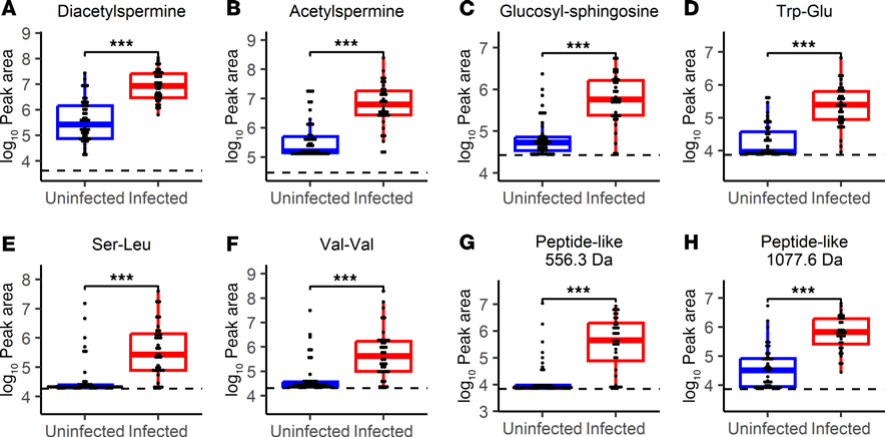
Seroma fluid features that differentiate infection status. Dot plots and box-and-whisker plots showing prevalence of (**A**) diacetylspermine, (**B**) acetylspermine, (**C**) glucosyl-sphingosine, (**D**) Trp-Glu, (**E**) Ser-Leu, (**F**) Val-Val, and (**G** and **H**) 2 unidentified peptide-like features in specimens collected at time of implant removal from uninfected (blue) and infected (red) breasts. Significance is denoted by FDR-adjusted (BH method) Wilcoxon’s rank sum *P* values (***P < 0.001). Exact FDR-adjusted *P* values may be found in Table 4. Box-and-whisker plots exclude outliers and depict the minimum and maximum values (whiskers), the upper and lower quartiles, and the median. Dashed lines indicate mean peak area in 5 aligned methanol blanks. As gap filling takes into account sample-specific background, some specimens may have calculated peak areas under this level.

To determine the chemical identities of these 16 infection biomarker candidates, we used accurate mass measurements and LC-MS/MS fragmentation patterns of seroma fluid features to search spectral libraries for matches. This yielded putative matches to acetylated polyamines, peptides, and a glycolipid (Table 4), consistent with metabolites originating in endogenous biochemical processes. The remaining features matched no database entries or were determined to be technical artifacts. Retention time and MS/MS spectrum comparison with reference standards identified the 2 polyamines as N1,N12-diacetylspermine and N1-acetylspermine (Figure 3, A and B, and Supplemental Figure 1). Likewise, the glycolipid matched the retention time and MS/MS spectrum of commercially available glucosyl-sphingosine and was distinguishable from its isomer, galactosyl-sphingosine, a component of myelin (Figure 3C and Supplemental Figure 2). Finally, we putatively identified multiple distinct peptide-like features, including Trp-Glu, Ser-Leu, and Val-Val (Figure 3, D–F), as well as 2 larger peptides (Figure 3, G and H) whose tandem MS/MS spectra contained similarities to di- or tripeptides from spectral libraries (Supplemental Figures 3 and 4). Small peptides may derive from many sources, including de novo production or specific or nonspecific proteolysis from larger polypeptides. Additionally, dipeptides may represent source decay fragments of larger peptides cleaved during ionization in the mass spectrometer and not necessarily the free dipeptide itself. Each identified biomarker candidate exhibited high predictive value, with AUCs of 0.82–0.91 (Table 4).

These infection-associated small molecules represent at least 3 categories of endogenously produced biomolecules. Increases in specific acetylated polyamines have been detected in the urine of pulmonary tuberculosis patients (diacetylspermine) and in gram-negative bloodstream infections (N-acetylputrescine and N1,N8-diacetylspermidine) (44–46). Spermidine/spermine acetyltransferase-1 (SAT1), an enzyme that produces N-acetylspermine, is implicated in neutrophil recruitment in a murine tumor model system (47–50). Glucosyl-sphingosine (lyso-GL1, lyso-Gb1) is the deacylated product of glucosylceramides that exhibit antimicrobial activity and are systemically elevated in patients with systemic inflammatory response syndrome (51–54). It may also be a by-product of excess lysosomal lipid degradation (55). While not as widely recognized in this context as cytokines, chemokines, or immune cells, these molecules are robustly associated with infection in the study cohort, implicate distinctive biochemical pathways, and plausibly play roles in immune cell engagement and host defense.

### Negative molecular correlates of infection.

Although the molecular features that are best able to differentiate infection status were positively associated with infection, several negatively associated features were also detected. In the set of 30 features decreased at least 4-fold with an FDR-adjusted *P* < 0.01, we identified 2 synthetic molecules: the antibiotic cefazolin (AUC = 0.71; Supplemental Figure 5) and the organic dye crystal violet (AUC = 0.76; Supplemental Figure 6). Eight additional negative correlates were strongly associated with cefazolin (Supplemental Table 2 and Supplemental Figure 5) and likely represent cefazolin metabolites or impurities. Two additional negative correlates were strongly associated with crystal violet (Supplemental Table 3 and Supplemental Figure 6) and had masses consistent with demethylated variants of crystal violet. The negative association between cefazolin and infection corresponds to the preferential use of cefazolin surgical prophylaxis in uninfected compared with infected patients at the time of implant removal (78.3% uninfected vs. 44.4% infected; Table 2). Crystal violet is present in surgical markers used at our institution, and their negative association with infection is attributable to more extensive markings used for planning second-stage reconstructive procedures in uninfected patients. While the post facto anthropogenic origin of these molecules places them outside our goal of tracking biomolecules derived from infection pathophysiology, these findings affirm our overall approach to identifying infection-associated molecules.

### Human defensins.

A canonical immune response at sites of infection is recruitment of neutrophils and other myeloid cells in response to local stimuli. To quantify this, we sought to measure human neutrophil peptides (HNP1–3; also known as α-defensins 1–3), a series of human myeloid cell defensins that are stored in neutrophil granules prior to release. Qualitative immunoassay-mediated detection of HNPs has been shown to be a marker of infection in prior studies of breast implant reconstruction (40, 41). Although the amphipathic defensins (~3.4 kDa) are larger than the peptides that emerged from our untargeted metabolomic analysis, they are strongly cationic, with multiple charge states that yield ions within the *m/z* window used for metabolite detection (32). We manually identified LC-MS features in seroma specimens with accurate masses consistent with HNP1–3 that had been filtered out by the peak quality filters (HNP2) or missed by the automated peak caller used for untargeted metabolomic profiling (HNP1 and HNP3). We further confirmed the identify of these distinctive LC-MS features by comparison with commercially available standards (Supplemental Figure 7).

**Figure 4:**
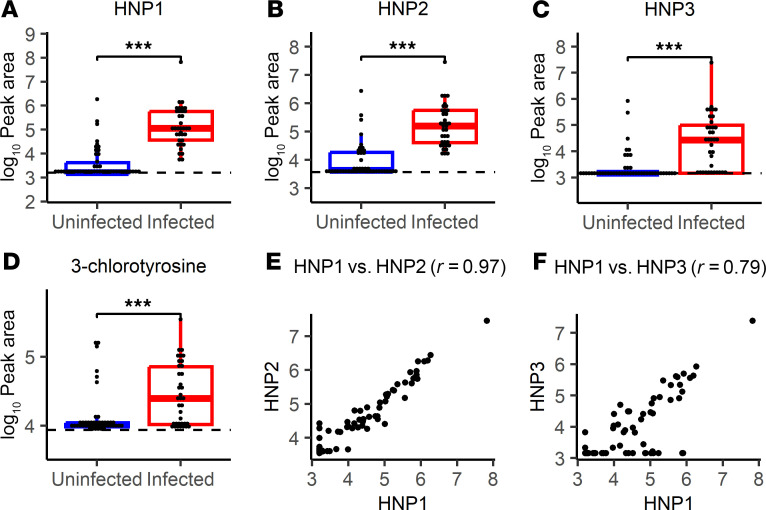
Neutrophil presence and activation markers in seroma fluid differentiate infection status. Dot plots and box-and-whisker plots showing prevalence of (**A**) HNP1, (**B**) HNP2, (**C**) HNP3, and (**D**) 3-chlorotyrosine in specimens collected at time of implant removal from uninfected (blue) and infected (red) breasts. (**E**) Dot plot showing relative abundance (log_10_ peak area) of HNP1 versus HNP2. (**F**) Dot plot showing relative abundance (log_10_ peak area) of HNP1 versus HNP3. Significance for targeted markers is denoted by unadjusted Wilcoxon’s rank sum *P* values (***P < 0.001). Box-and-whisker plots exclude outliers and depict the minimum and maximum values (whiskers), the upper and lower quartiles, and the median. For HNP2 and 3-chlorotyrosine, dashed lines indicate mean peak area in 5 aligned methanol blanks. As gap filling takes into account sample-specific background, some specimens may have calculated peak areas under this level. For HNP1 and HNP3 (manually integrated peaks), dashed lines indicate lowest detected abundance in any fluid sample.

HNP1 (Figure 4A) and HNP2 (Figure 4B) were highly associated with infection (AUCs of 0.93 and 0.92, respectively). The HNP3 AUC was lower (0.78; Figure 4C), likely because 10%–37% of humans lack HNP3 (56). HNP1 and HNP2 levels were highly correlated (Figure 4E), while HNP3 and HNP1 levels exhibited greater deviation (Figure 4F). HNP1 and HNP2 thus exhibit a quantitative association with infection comparable with those for the acetylated polyamines, glucosyl-sphingosine, and other peptide-like features identified above.

### Association with neutrophil activation.

Because HNPs are prepackaged in cellular vesicles, elevated levels may indicate the presence of myeloid cells but not their degree of activation in response to inflammatory stimuli. To identify neutrophil and monocyte activation, we sought to quantify 3-chlorotyrosine, 3-bromotyrosine, and 3-nitrotyrosine, modified amino acids generated during the respiratory burst when azurophil granules release myeloperoxidase, which generates reactive species through oxidation of chloride, bromide, or nitrite (derived from nitric oxide) ions (57–62). We detected features matching retention times and MS/MS spectra of commercially available reference standards of 3-chlorotyrosine and 3-bromotyrosine (Supplemental Figure 8), but not 3-nitrotyrosine. These features were filtered out by our peak quality filters during initial untargeted analysis. Each was moderately associated with infection status, with AUCs of 0.72 and 0.68 for 3-chlorotyrosine and 3-bromotyrosine, respectively (Figure 4D and Supplemental Figure 8). Of these, 3-chlorotyrosine is the most specific marker of myeloperoxidase activity, which is distinguished from other human peroxidases by its ability to generate chlorinating species (58). 3-Bromotyrosine may be produced by myeloperoxidase or eosinophil peroxidase (59, 63). These results are consistent with a neutrophil cell activation sufficient to stimulate both the respiratory burst and azurophil granule release.

### Infection-associated molecules precede infection diagnosis.

**Figure 5:**
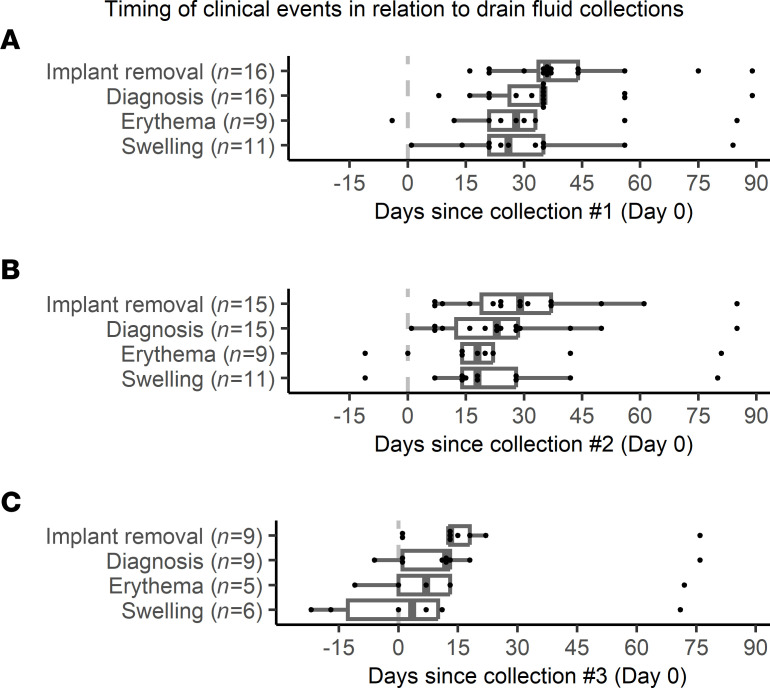
Timing of clinical events in relation to drain fluid collections. Dot plots and box-and-whisker plots showing the temporal relation of first recorded instance of erythema, first recorded instance of swelling, infection diagnosis, and implant removal in longitudinal drain fluid specimens from breasts in cohort 2 that were removed due to infection (*n* = 16). (**A**) First (*n* = 16), (**B**) second (*n* = 15), and (**C**) third (*n* = 9) drain fluid collections. All patients had an initial drain fluid collection, but drain removal resulted in dropouts for further time points. Dashed lines at day 0 indicate the date the drain fluid was sampled. Positive values indicate that implant removal, infection diagnosis, first recorded instance of erythema, and/or first recorded instance of swelling occurred after the drain fluid collection. Box-and-whisker plots exclude outliers and depict the minimum and maximum values (whiskers), the upper and lower quartiles, and the median.

We hypothesized the existence of a subclinical infectious state preceding implant infection diagnoses. To test this, we measured the infection correlates identified above in longitudinal surgical drain fluid specimens from 44 breasts in 32 patients in cohort 2. Specimens were obtained at the first 3 clinical follow-up visits for 44, 39, and 17 breasts, respectively, with dropouts due to prior drain removal. Visits 1–3 occurred a median of 10, 20, and 27 days after mastectomy with immediate breast implant reconstruction. For the 16 breasts progressing to infection, the timing of drain fluid collection in comparison with implant removal, infection diagnosis, the first recorded instance of erythema, and the first recorded instance of swelling can be found in Figure 5 and Supplemental Table 4. In infected breasts, drain fluid collections on visits 1–3 occurred a median of 36, 29, and 13 days prior to implant removal, respectively. Specimen collections on visits 1–3 preceded infection diagnoses in 16/16, 15/15, and 8/9 breasts (median 35, 23, and 12 days prior, respectively). Notably, 7 of the 16 breasts were only formally diagnosed with infection at the time of implant removal. For infected breasts that developed overt erythema or swelling, specimen collections on visits 1–3 preceded erythema in 8/9, 7/9, and 3/5 breasts (median 28, 18, and 7 days prior to documentation of erythema, respectively) and preceded swelling in 11/11, 10/11, and 3/6 breasts (median 26, 18, and 3.5 days prior to documentation of swelling, respectively). These longitudinal specimens thus sample the postoperative period prior to infection diagnoses and recorded symptom onset.

**Figure 6:**
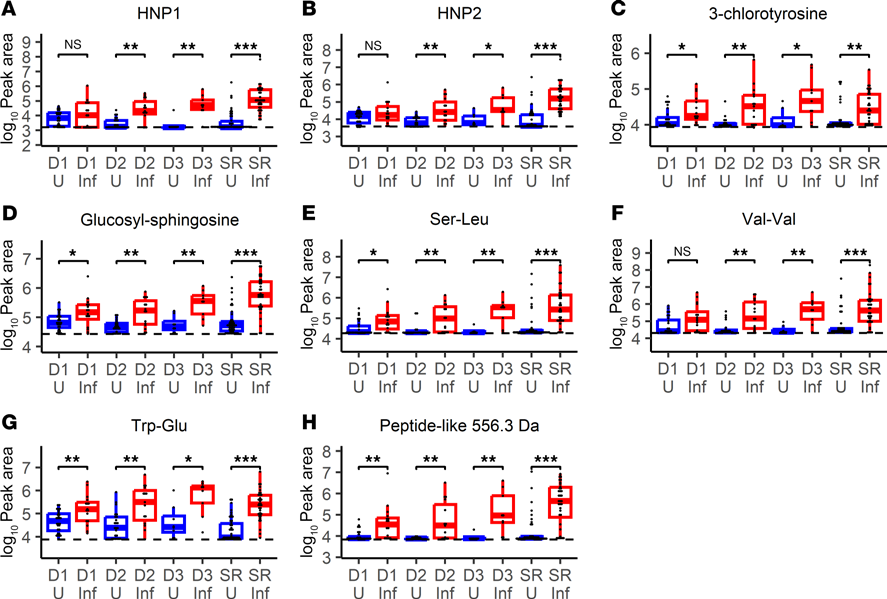
Infection biomarkers in longitudinal drain fluid collected before implant removal. Dot plots and box-and-whisker plots showing prevalence of (**A**) HNP1, (**B**) HNP2, (**C**) 3-chlorotyrosine, (**D**) glucosyl-sphingosine, (**E**) Ser-Leu, (**F**) Val-Val, (**G**) Trp-Glu, and (**H**) peptide-like 556.3 Da biomarkers from longitudinally collected drain fluid in cohort 2 (D1, drain collection 1; D2, drain collection 2; D3, drain collection 3) and seroma fluid collected from breasts at the time of implant removal (SR) in breasts from both cohorts. Specimens are additionally stratified on whether they were from breasts with implants removed due to infection (Inf, red) or remaining uninfected (U, blue). Significance is denoted by FDR-adjusted (BH method) Wilcoxon’s rank sum *P* values (*P < 0.05, **P < 0.01, ***P < 0.001). Exact FDR-adjusted *P* values may be found in Table 5. Box-and-whisker plots exclude outliers and depict the minimum and maximum values (whiskers), the upper and lower quartiles, and the median. Dashed lines indicate mean peak area in 5 aligned methanol blanks. As gap filling takes into account sample-specific background, some specimens may have calculated peak areas under this level. For HNP1 (manually integrated), the dashed line indicates lowest detected abundance in any fluid sample.

At the first postoperative clinic visit, breasts with TEs subsequently removed due to infection exhibited significant (all *P* < 0.05) but modest (mean 1.7- to 3.9-fold increase, AUC 0.71–0.75) elevations of 3-chlorotyrosine, glucosyl-sphingosine, Trp-Glu, Ser-Leu, and the 556.3 Da peptide-like molecule compared with breasts that did not develop infection (Figure 6 and Table 5). At the second postoperative clinic visit, these differences were sustained for 3-chlorotyrosine (mean 3.0-fold increase), glucosyl-sphingosine (mean 3.0-fold increase), Trp-Glu (mean 7.8-fold increase), Ser-Leu (mean 4.0-fold increase), and the peptide-like molecule at 556.3 Da (mean 6.2-fold increase) and additionally included HNP1 (mean 8.4-fold increase), HNP2 (mean 4.6-fold increase), and Val-Val (mean 6.3-fold increase) with AUCs ranging from 0.77 to 0.83. These markers remained significant at visit 3 (all *P* < 0.05, mean 4.0- to 21.7-fold increase, AUC 0.81–0.97; Figure 6 and Table 5), although third drain collections were only available for 17/44 breasts. Acetylated spermines and the 1,077.6 Da peptide-like molecule were not consistently different between infected and uninfected breast groups (Supplemental Figure 9 and Table 5). These findings are consistent with the existence of a subclinical infection state preceding implant infection diagnoses, indicated by multiple inflammation-associated molecules detectable in surgical drain fluid.

### Bacteria preceding infection diagnosis.

The presence of increased host-derived infection-associated metabolites in drain fluids from patients who proceed to clinical infection raises the possibility that these patients had subclinical infections at the earlier time points. To assess this possibility, we sought to determine whether the specific bacterial species associated with infection at time of implant removal was also present in preceding drain fluid cultures. The microbiology of drain fluid cultures was diverse (Supplemental Table 5), although *P*. *aeruginosa* (*n* = 17/40 drain collections), coagulase-negative *Staphylococcus* (*n* = 10/40 drain collections), and *Enterococcus faecalis* (*n* = 11/40 drain collections) were the most common organisms cultured from drain fluid specimens from breasts progressing to infection. Coagulase-negative *Staphylococcus* (*n* = 26/60 drain collections) and *E*. *faecalis* (*n* = 12/60 drain collections) were the most common organisms cultured from drain fluid specimens from breasts that did not progress to infection.

Due to the diversity of organisms isolated from drains, we limited our comparison of bacterial presence in drain fluids compared with presence at the time of implant removal to the 9 bacteria isolated from at least 4 separate drain fluid collections (Supplemental Table 6). In cohort 2, 100% (7/7) of breasts that later developed *P*. *aeruginosa* infection grew *P*. *aeruginosa* in at least 1 preceding drain fluid collection, compared with 2/37 (5.4%) breasts that remained uninfected (*n* = 1) or had non–*P*. *aeruginosa* infections (*n* = 1) (*P* < 0.001, 2-tailed Fisher’s exact test). *Corynebacterium jeikeium, Serratia marcescens, Staphylococcus warneri, Enterobacter spp., Staphylococcus haemolyticus, S*. *aureus, E*. *faecalis,* and *Staphylococcus epidermidis* had no significant association (2-tailed Fisher’s exact test) between presence in at least 1 drain and infection at time of implant removal (Supplemental Table 6). In this cohort, *P*. *aeruginosa* recovery from a drain was uniquely associated with subsequent *P*. *aeruginosa* breast infection. This is consistent with subclinical infections, though we cannot rule out the possibility that *P*. *aeruginosa* in drains is a colonizer, the presence of which predicts subsequent infection.

### Bacterial exometabolites precede P. aeruginosa infection diagnosis.

**Figure 7:**
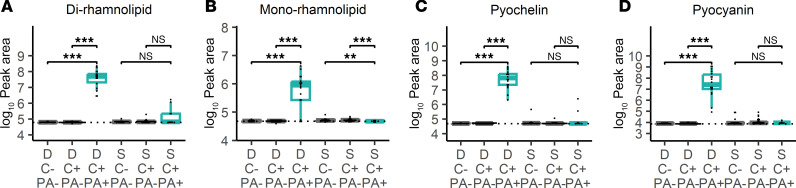
*P*. *aeruginosa* metabolites in longitudinal drain fluid and seroma fluid samples. Dot plots and box-and-whisker plots showing prevalence of (**A**) dirhamnolipid, (**B**) monorhamnolipid, (**C**) pyochelin, and (**D**) pyocyanin in both surgical drains (D) and specimens collected at time of implant removal (S) with specimens that were culture positive (C+) or culture negative (C–). Culture positive samples are further differentiated into samples that did (PA+) or did not (PA–) grow *P*. *aeruginosa*. Significance is denoted by unadjusted Wilcoxon’s rank sum *P* values (**P < 0.01, ***P < 0.001). Box-and-whisker plots exclude outliers and depict the minimum and maximum values (whiskers), the upper and lower quartiles, and the median. Dashed lines indicate mean peak area in 5 aligned methanol blanks. As gap filling takes into account sample-specific background, some specimens may have calculated peak areas under this level.

The infection metabolome may also include species-specific bacterial exometabolites secreted during infection. Given the high concordance of *P*. *aeruginosa* presence in drains and future *P*. *aeruginosa* infection, we sought to determine if the well-documented *P*. *aeruginosa* exometabolites pyocyanin, dirhamnolipid, monorhamnolipid, and pyochelin (64, 65) were present in the 18 drain specimens positive for *P*. *aeruginosa*. These metabolites were identified by retention time and MS/MS spectral matches with authentic standards (Supplemental Figure 10 and Supplemental Table 7). Pyocyanin, *P*. *aeruginosa* surfactants (mono- and dirhamnolipids), and the metallophore pyochelin were significantly elevated in drain fluid specimens from which *P*. *aeruginosa* was cultured, relative to specimens from which only non–*P*. *aeruginosa* species or no bacteria were cultured. The 3 highest intensity metabolites (dirhamnolipid, pyochelin, and pyocyanin) were present in 18/18 (100%) of *P*. *aeruginosa* culture-positive drain fluid specimens (Figure 7). These exometabolites were less frequently detected (pyocyanin, 0/9 samples; dirhamnolipid, 4/9 samples; pyochelin, 2/9 samples) in operative seroma fluid specimens that grew *P*. *aeruginosa* (8 implants removed due to infection; 1 implant removed due to noninfectious complications) (Figure 7) possibly because the microbial load had been reduced by antibiotic treatment prior to implant removal. Together, these findings suggest that culture-independent *P*. *aeruginosa* detection through its distinctive exometabolites may help inform diagnosis and treatment decisions in patients with suspected breast implant–associated infections.

## Discussion

In this study, we sought small molecules that identify implant infections in patients undergoing postmastectomy breast reconstruction. This effort yielded a diverse set of canonical and noncanonical small-molecule infection correlates in seroma fluid from sites of infection. These included a sphingolipid, acetylated polyamines, neutrophil defensins, halogenated tyrosines, and unidentified peptide-like molecules, each with plausible host origins connected to inflammation. A subset of these metabolites were elevated in drain fluid specimens collected prior to infection diagnosis and appeared before, or at comparable times to, onset of compatible clinical symptoms. In addition, *P*. *aeruginosa*, and the distinctive infection-associated molecules it secretes, were recovered in specimens prior to diagnosed *P*. *aeruginosa* infections at early time points, raising the possibility of subclinical infections in these patients. The existence of such infections, and diagnostic signatures to identify them, raise the prospect of new preemptive treatment strategies for implant infections to improve patient outcomes.

The infection-associated molecules identified here plausibly originate from several distinctive biological sources. As shared correlates of infections in multiple patients with different pathogens, these molecules are likely to derive from general human responses to infection. Acetylated spermine (and spermidine) are known products of mammalian SAT1, the expression of which has been associated with immune cell recruitment in both cancer and a variety of infections (47–50). Acetylated polyamines have been found to be diagnostic markers in other infections, including pulmonary tuberculosis and bloodstream infections (44–46). Elevations in HNP1–3 are consistent with increased recruitment of neutrophils, which transport these defensins from blood to tissue within prepackaged granules (66). HNP1 detection has previously shown high sensitivity and specificity in diagnosing orthopedic infections (67) and breast implant reconstruction infections (40–42) in prior studies. The metabolite 3-chlorotyrosine is likely formed in situ in tissue when myeloperoxidase-derived reactive chlorine species generated during the respiratory burst of activated neutrophils chlorinate tyrosine (57–62). Glucosyl-sphingosine may represent yet another antimicrobial product at sites of infection that is generated in response to infection or may be a by-product of excess lysosomal lipid degradation by macrophages (51–55). This glycolipid is generated by acid ceramidase cleavage of glucosylceramide substrates to release the corresponding glucosyl-sphingosine, which can act as a cationic surfactant with broad spectrum antibacterial activity (53, 54). The infection-associated, nondefensin peptides identified in this study are of unclear origins, though it is possible they represent products of the various proteases released during inflammation (66). Together, these molecules plausibly correspond to markers of immune cell recruitment, activation, and antibacterial effector release during the human inflammatory response. An optimal future clinical assay might define a diagnostic signature composed of multiple markers both to represent the pathophysiologic process and to avoid false positive results from isolated elevations in a single marker.

These molecular signatures were discovered using specimens collected directly from the breast pocket at the time of implant removal. Paramount to the clinical utility of these signatures is whether they are present and detectable early enough to meaningfully inform an actionable therapeutic strategy that prevents TE explantation. Several of these markers, including 3-chlorotyrosine, glucosyl-sphingosine, HNP1, HNP2, and other peptide-like markers, were significantly increased in drain fluid from breasts that ultimately developed overt evidence of infection necessitating implant removal, most prominently in drain fluids from the second and third clinic visits. Notably, the second clinic visit, at a median time of 20 days after reconstruction, preceded infection diagnosis in all infected breasts (median of 23 days prior to formal diagnosis), suggesting that an underlying subclinical infection process may be present before definitive clinical manifestations. A future study might assess the predictive characteristics of a prospectively applicable diagnostic fluid assay obtained shortly (15–20 days) after implant placement, with the ultimate goal of using this to guide more effective, targeted therapeutic approaches to patients at high risk of infection.

The possibility of a subclinical inflammatory process that precedes diagnosed clinical infection may be an important consideration in future implant infection studies. One explanation of infection development is that bacterial seeding, potentially at time of implantation, causes this subclinical inflammatory process as the nascent infection develops. This would be consistent with findings from the orthopedic field, where early infection weeks to months after implant placement is often thought to be due to bacterial seeding during the initial surgery (9, 10). Another possibility in the case of breast reconstruction is that normal postsurgical inflammation fails to resolve, resulting in tissue damage that predisposes a breast to bacterial colonization and infection from external sources such as drains. The breast is also a clean-contaminated surgical site, chronically colonized by microbiota (68–71). Even with ideal surgical and sterile technique, it cannot be disregarded that bacteria-laden skin and ducts are disrupted in every mastectomy (68–71). Future studies will be required to further characterize the early pathophysiology of breast implant reconstruction infection.

There are several limitations to our study. This was a small, single-center study that lacked an external validation cohort. Follow-up studies are required to determine whether biomarkers identified in this study are reliable in other patient populations. In addition, our recruitment strategy for cohort 2 was limited to women at high risk of infection to maximize the number of specimens obtained in women with breast implant infection. While our primary analysis included patients from both cohorts, results from longitudinal drain fluid collections were derived exclusively from cohort 2, which may not be generalizable to lower-risk populations. Antibiotics were often administered before specimens were collected at implant removal, which may affect bacterial burden and metabolite secretion. Additionally, our definition of infection required the patient to satisfy the CDC definition of infection, which may not match definitions in other research studies that used alternative or unstated definitions of infection. It is possible that specimens exhibiting apparent false positive biomarker values represent patients (particularly those with tissue necrosis or wound dehiscence) on a trajectory toward CDC-defined infection that was never realized because they underwent curative implant removal before that definition was satisfied. Finally, longitudinal data in our study relied on noninvasive measurements of drain fluid collected at convenience samplings during routine clinical visits. Ideal samples for microbiological and metabolomic comparisons would be fluid collected aseptically from the breast pocket, as there are likely differences between fluid collected from the breast pocket and fluid collected in drains. To this end, available dual-port TEs that facilitate aspiration of periprosthetic fluid in the absence of a drain could be utilized in the future (72, 73). Overall, this preliminary pilot study lays the groundwork for future studies into the diagnosis, management, and pathophysiology of breast implant reconstruction infection.

The appropriate administration of antibiotics following breast implant reconstruction represents a major challenge in the management of breast cancer patients. Some evidence supports only a perioperative or short course of systemic antibiotic administration after implant reconstruction (74–76). Yet, the unacceptably high rates of infection that lead to compromised oncologic care, psychological distress, and socioeconomic burden have a powerful impact on physician prescribing behaviors and patient wishes that often lead to prolonged and empiric antibiotic use. Identification of molecular signatures that predict infection versus a quiescent clinical course could inform targeted antibiotic prescription patterns, facilitate responsible antibiotic stewardship, and promote surgeon and patient confidence.

## Methods

### Sex as a biological variable.

All patients in this study were women, consistent with all patients seeking breast reconstructive surgery at our institution. The degree to which findings are relevant to males is unknown.

### Cohort 1.

From September 2018 to January 2020, women 21 years and older who had previously undergone mastectomy and immediate or second-stage implant-based reconstruction (TEs or permanent implants) were recruited at the time of implant removal due to infectious complications, noninfectious complications, or removal of TEs at the time of second-stage reconstruction (Figure 1). TEs/implants were swabbed for bacterial growth, and seroma fluid in the breast pocket (if any) was collected and processed for microbial culture in the laboratory within 24 hours of collection. After inoculation of agar and liquid media in the laboratory, residual seroma fluid was aliquoted and frozen at –80°C for future metabolomics analysis.

### Cohort 2.

From April 2022 to July 2023, women 21 years and older undergoing mastectomy with immediate TE breast reconstruction with 1 or more risk factors for implant-associated infection (obesity, diabetes, or tobacco use) (77) were recruited prior to mastectomy or at their first plastic surgery clinic visit after mastectomy (Figure 1). All patients undergoing mastectomy had surgical drains placed to drain the breast pocket, which remained in situ until removal at the surgeon’s judgment (most often 1–3 weeks after mastectomy). At each postoperative clinic visit while drains were in place, samples of drain fluid were collected and processed for microbial cultures within 5 hours of collection. Residual drain fluid was aliquoted and stored frozen at –80°C for future metabolomics analysis. When expanders were removed for any reason, the removed TE was swabbed for bacterial growth, and seroma fluid in the breast pocket (if any) was cultured on agar plates and liquid media as above. Residual seroma fluid was aliquoted and frozen at –80°C for future metabolomic analysis.

### Definition of infection.

Breasts were considered infected if they met any of the following criteria, adapted from the CDC definition of surgical site infection (13, 14): (a) diagnosis by a surgeon, (b) purulent drainage or evidence of abscess, (c) positive aseptically collected culture at time of implant removal, or (d) implant removal with 2 of more of the following: incisional pain/tenderness or localized swelling; erythema; warmth; and/or fever > 38°C.

### Specimen culture and identification.

Samples were transported from the operating room to laboratory space at room temperature to maximize bacterial viability in culture. All culture work took place prior to freezing samples for metabolomics analysis. For drain and cohort 2 seroma fluid samples, 25 μL of fluid was cultured upon each of the following agar types (purchased from Hardy Diagnostics) and incubated at 37°C: HardyChrom UTI chromogenic medium, HardyChrom urine biplates, 5% sheep’s blood agar, Middlebrook 7H11 agar, and Brucella blood agar (with hemin and vitamin K) plates. For cohort 1, the same media as above were used except for use of MacConkey’s agar instead of the HardyChrom plates. Aerobic medium excluding Middlebrook agar was incubated for up to 3 days, anaerobic media (Brucella blood agar) up to 2 weeks, and Middlebrook agar up to 4 weeks. In addition, 25 μL of fluid was used to inoculate BacT/ALERT FA Plus and FN Plus blood culture bottles (cohort 1) or Brain Heart Infusion broth (Hardy Diagnostics; cohort 2), which was subcultured onto blood agar if other media were negative for growth. Colonies that were morphologically distinct were isolated and subcultured to obtain pure colonies, at which point they were saved in glycerol stocks at –80°C.

Implants were swabbed vigorously with 2 E-swabs. 25 μL of E-swab fluid was then cultured on each media type in the same manner as described above. Individually distinct colonies that were morphologically different were isolated. Specimens collected at the time of implant removal were considered positive for an organism if they grew either from seroma fluid and/or the swabbed implant. In cases where the amount of seroma fluid obtained in the operating room was not sufficient for both metabolomics and culture, metabolomic experiments were prioritized and the seroma fluid was not cultured. In these patients, culture results came only from the implant swabs (*n* = 7). All bacterial identification of organisms from both cohorts was performed using MALDI-TOF MS with the Vitek MS v2.0 system (bioMérieux) as previously described (78–82).

### Specimen storage and preparation for LC-MS.

After microbial cultures were performed, residual fluid was stored at –80°C when not in use. To prepare for metabolomic analysis, drain and intraoperatively collected seroma fluids were methanol extracted according to a modified procedure (83). 400 μL of ice-cold methanol was added to 100 μL of sample, mixed by vortexing for 30 seconds, and incubated on ice for 20 minutes before centrifugation at 20,000*g* for 5 minutes. The liquid fraction of the extract was then filtered using PVDF filter plates and placed in appropriate sample vials for MS.

### Untargeted metabolomic profiling.

Samples were analyzed using a Thermo Orbitrap ID-X mass spectrometer coupled with a Vanquish UHPLC system (Thermo Fisher Scientific) using a reverse-phase Acquity HSS T3 column (Waters) according to a previous procedure (84) with the following modification: samples were run separately in positive mode and negative mode with MS1 Orbitrap mass resolving power set to 120,000. MS2 spectra were obtained using the AcquireX protocol (Thermo Fisher Scientific) on a pooled mixture of samples according to a previous procedure (84). If additional spectra of a feature were required for identification, MS2 spectra were obtained from pooled subsets of samples containing the feature in subsequent targeted LC-MS/MS runs. Peak detection, alignment, and integration were performed in Compound Discoverer 3.3 (Thermo Fisher Scientific). In samples where a metabolite was not detected, the software estimated the area of the largest theoretical Gaussian peak indistinguishable from the spectrum noise in the extracted ion chromatogram where the peak was expected (gap filling). In the case of HNP1 and HNP3, the software failed to integrate peaks that we manually confirmed using reference standards to be these defensins (Supplemental Figure 7 and Supplemental Table 7). Peak areas for these compounds were manually integrated in xcms (85–87) from the most abundant isotopolog, and the limit of detection was estimated as 0.001 below the minimum integrated log_10_ peak area in any sample.

### Preprocessing of the untargeted dataset for primary analysis.

Features were only retained for untargeted analysis if they had a peak quality rating of 5/10 (calculated by Compound Discoverer 3.3) in at least 10 samples of the 82 samples included in primary untargeted analysis, as we did not expect compounds present in less than 10/82 samples to be diagnostically useful. The software used for peak detection attempts to remove isotopologs and adducts and retain only the highest intensity adduct/isotopolog. However, like all automated peak-calling software, Compound Discoverer remains imperfect at this task (88). In addition, the version 3.3 software does not account for potential in-source fragmentation artifacts, which historically have been less studied than isotopes or adducts (89). To minimize the effect of these artifacts on our dataset, we created a network of highly correlated features with edges between features with both > 0.9 conditional correlation and retention times within 0.1 minutes of each other. We retained only the most intense feature for each connected component of the network. Conditional correlation was defined as Pearson’s correlation between features among the 82 samples in our primary analysis excluding samples where Compound Discoverer gap-filling status was filled by spectrum noise for either sample. An annotated example and visual explanation of one of these networks are provided in Supplemental Figure 11. This preprocessing resulted in the 2,540 features used for untargeted analysis.

### Prioritization of features with DCV.

Initially in our analysis, we were uncertain whether our samples might be subject to unknown dilution effects due to the potentially multifactorial origin (serum/blood leakage, interstitial fluid, inflammatory infiltrate, etc.) of our intraoperative seroma fluid samples. In such a scenario, metabolomic data must be treated as compositional and analyzed accordingly. We opted to use the DCV scores calculated in SelEnergyPerm (43), a compositional data analysis pipeline, to identify features that retained information content regardless of possible unknown dilution factors. The DCV scores are calculated across all possible pairwise log ratios between features within a given dataset to form an aggregate test statistic from several parametric and nonparametric tests for each log ratio. Summing all log ratios associated with a given feature gives its DCV score, with higher values indicating features that have significant information content when normalized to many features within a given sample. To avoid potential outlier samples strongly affecting DCV scores for features, we performed 25 repeats of randomized 75% subsampling upon our dataset where we calculated DCV scores for features using only those samples. We then selected features that were consistently within the top 20 rank of DCV scores in at least 50% of our subsamples, showing that they had information content robust to sample dropouts and potential outliers. This analysis resulted in 16 features that were then prioritized for identification.

### Feature identification.

To identify features of interest, MS2 spectra were acquired either by the AcquireX algorithm, as previously described (84), or by subsequent targeted LC-MS/MS runs. MS2 spectra were compared with the mzCloud (Thermo Fisher Scientific) and the GNPS public spectral libraries (90, 91), ALL_GNPS_NO_PROPOGATED.mgf, which includes MassBank (92) libraries, downloaded on July 31, 2024. Comparisons with the mzCloud library were done using the open-access web client at https://www.mzcloud.org/ Comparisons with GNPS/MassBank databases were performed using the matchms (93) python package implemented in R using the reticulate (94) package. Spectra were preprocessed to remove peaks within 1 Da of the precursor *m*/*z*, and modified cosine scores and numbers of fragment matches were calculated using a tolerance of 0.02 Da. Spectral matches with modified cosine scores > 0.7 and 6 or more fragment matches were manually examined to determine the most likely compound identities. Chemical standards used to confirm compound identities can be found in Supplemental Table 7. Supplemental Table 7 also lists supplemental figures showing retention time and MS/MS spectra comparisons of metabolite features and reference standards. Supplemental Table 8 contains a similar list of supplemental figures for compounds that were putatively identified by comparison with MS libraries alone.

### Statistics.

All statistical analyses were performed in R (95). Heatmap and cluster analysis were performed using the ComplexHeatmap (96, 97) and pheatmap (98) packages. For volcano plots, *P* values were calculated using Wilcoxon’s rank sum test and adjusted for FDR using the BH method upon the 2,540 compounds in the primary untargeted dataset. Multivariate compositional analysis was performed with SelEnergyPerm (43) as described above. AUCs were calculated in R using the pROC (99) package. Unless otherwise stated, all pairwise statistical comparisons between sample groups were performed with Wilcoxon’s rank sum test, and all FDRs were calculated with the Benjamini-Hochberg method.

### Study approval.

Approval was obtained from the Washington University Institutional Review Board with written informed consent for cohort 1 (IRB 20170714) and cohort 2 (IRB 202111145).

### Data availability.

Code generated as part of this study can be found at GitHub (https://github.com/jawildenthal/implant_metabolomics; commit ID 17ccb0e54f5f7f9801045d1d146af7c70696eea0). Values for data points in graphs are reported in the Supporting Data Values file. Primary LC-MS data in Thermo RAW format are available upon request.

## Author contributions

JPH, MAO, DKW, and TMM conceptualized and designed the study. TMM, KEB, MMT, JMC, THT, JMS, and RAA contributed to clinical design and interpretation. JAW, JIR, and HF performed MS analyses. JAW, HDT, and MAO performed microbiological characterization of samples. TMM, KEB, MMT, JMC, THT, JMS, and RAA collected specimens. JAW, MAO, and HDT processed specimens. JAW, MAO, JPH, DKW, and TMM wrote the manuscript. JIR and PJM edited the manuscript. JAW, JIR, and PJM performed statistical analyses. MAO and KBN conducted chart review and coordination of patients. All authors critically reviewed the manuscript.

## Funding support

This work is the result of NIH funding, in part, and is subject to the NIH Public Access Policy. Through acceptance of this federal funding, the NIH has been given a right to make the work publicly available in PubMed Central.

- CDC cooperative agreement U54CK000609 to Victoria J. Fraser.
- Institute for Clinical and Translational Science Award UL1TR002345 to William J. Powderly.
- NCI Cancer Center Support Grant P30 CA091842 to Timothy Eberlein.
- National Institute of Allergy and Infectious Diseases training grant T32AI007172 to Daniel Goldberg supported JAW.
- National Institute of Diabetes and Digestive and Kidney Disease (NIDDK) awards RO1 DK125860 and RO1 DK111930 to JPH.
- NIDDK award P30 DK020579 to Clay Semenkovich.

## Supplementary Material

Supplemental data

ICMJE disclosure forms

Supporting data values

## Figures and Tables

**Table 5:**
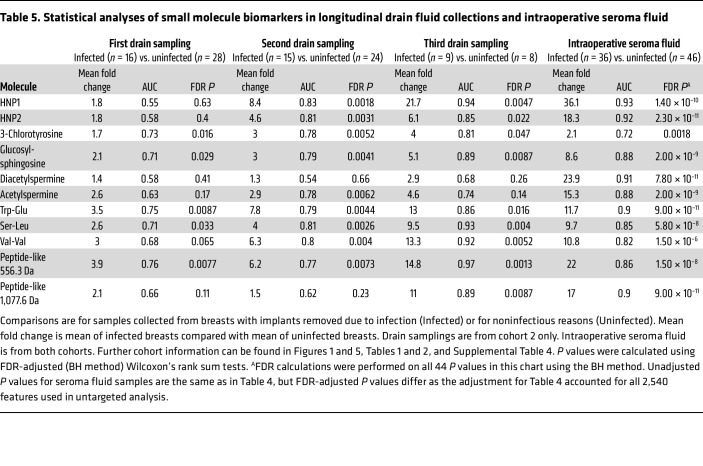
Statistical analyses of small molecule biomarkers in longitudinal drain fluid collections and intraoperative seroma fluid

**Table 4:**
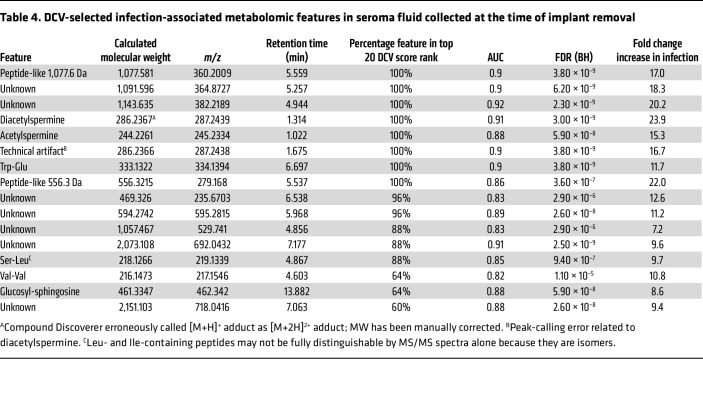
DCV-selected infection-associated metabolomic features in seroma fluid collected at the time of implant removal

**Table 2:**
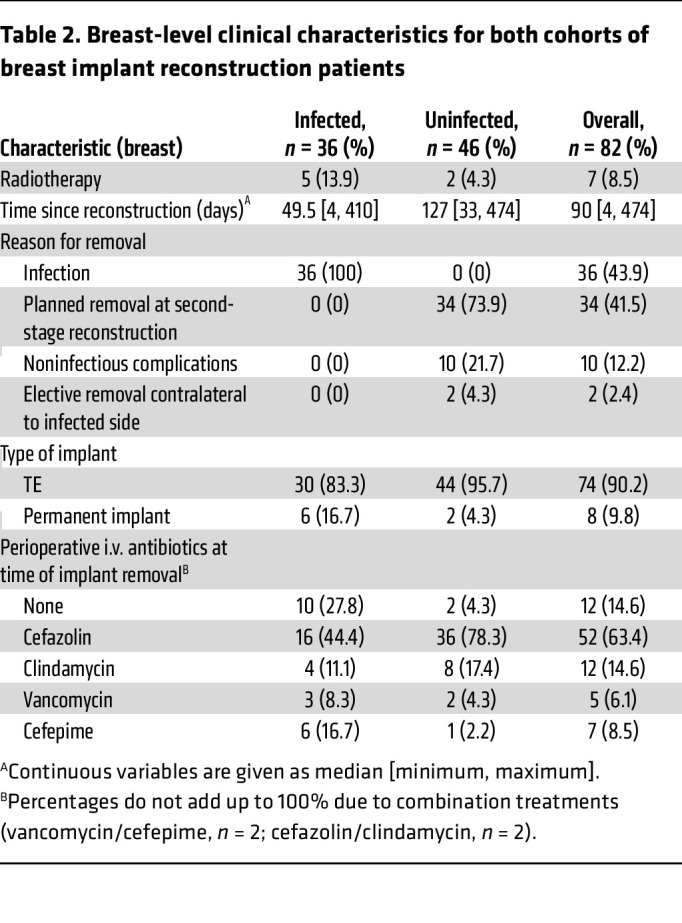
Breast-level clinical characteristics for both cohorts of breast implant reconstruction patients

**Table 1:**
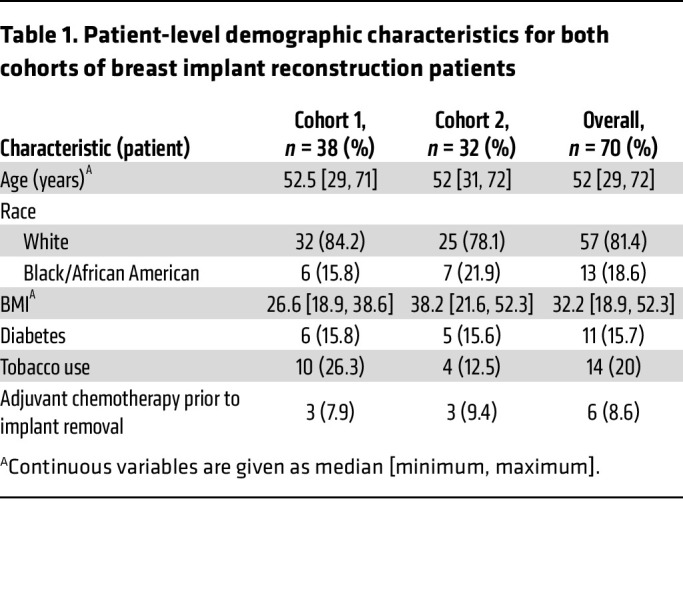
Patient-level demographic characteristics for both cohorts of breast implant reconstruction patients

**Table 3:**
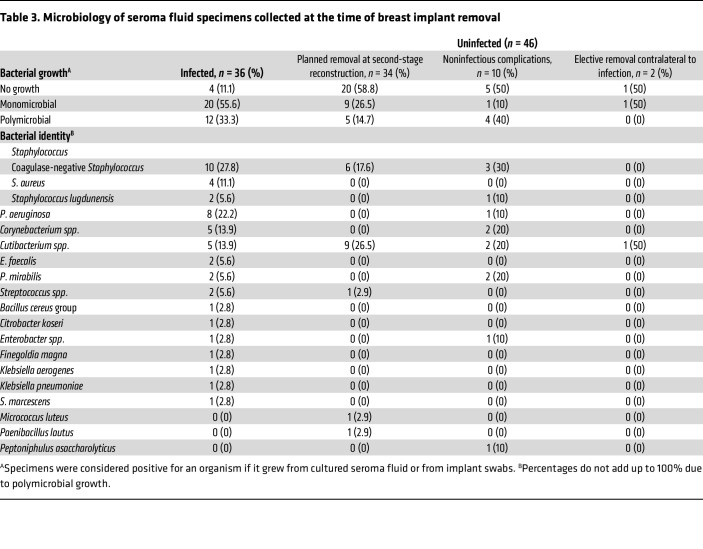
Microbiology of seroma fluid specimens collected at the time of breast implant removal

## Version 1 12/23/2025

In-Press Preview

## Version 2 02/16/2026

Electronic publication
