# Supplementary material for: Small-molecule correlates of infection precede infection diagnosis in breast implant reconstruction patients
Source: J Clin Invest. 2025 Dec 23;136(4):e192104. doi: 10.1172/JCI192104 (PMC12904714; doi:10.1172/JCI192104)
Supplement: Supplemental data [file jci-136-192104-s073.pdf]

TABLE OF CONTENTS (SUPPLEMENT)

Figure S1: Mass spectrometric identification of diacetylspermine and acetylspermine ... 2

Figure S2: Mass spectrometric identification of glucosyl-sphingosine 3

Supplemental methods (Resolution of glucosyl vs galactosyl sphingosine) 4

Figure S3: Mass spectrometric library matches of putative dipeptide features............. 5-6

Figure S4: Mass spectrometric library matches of putative peptide-like features......... 7-8

Figure S5: Identification of cefazolin and associated features in seroma fluid samples collected at time of implant removal 9

Figure S6: Identification of crystal violet and associated features in seroma fluid samples collected at time of implant removal 10-11

Figure S7: Mass spectrometric identification of HNP1-3.. 12-15

Figure S8: Mass spectrometric identification of 3-chlorotyrosine and 3-bromotyrosine 16-17

Figure S9: Biomarkers of infection that are not consistently increased in longitudinal drain fluid samples collected prior to implant removal in patients progressing to infection 18

Figure S10: Mass spectrometric identification of *Pseudomonas aeruginosa* metabolites 19-21

Figure S11: Example of a single putative molecule giving rise to multiple features....... 22

Table S1: Microbiology of seroma fluid specimens collected at the time of breast implant removal separated by mono- and poly-microbial culture 23

Table S2: Features decreased in infection that are highly correlated with cefazolin...... 25

Table S3: Features decreased in infection that are highly correlated with crystal violet 26

Table S4: Table of clinical events in relation to drain fluid collections in all breasts from cohort2 27

Table S5: Microbiology of drain fluid collections #1-3 in cohort2................................... 28

Table S6: Concordance of bacterial growth in drains with future infection with the same bacterium for the nine most prevalent bacteria in drains 29

Table S7: Compounds identified by standards.. 30

Table S8: Compounds identified by comparisons to library spectra 31

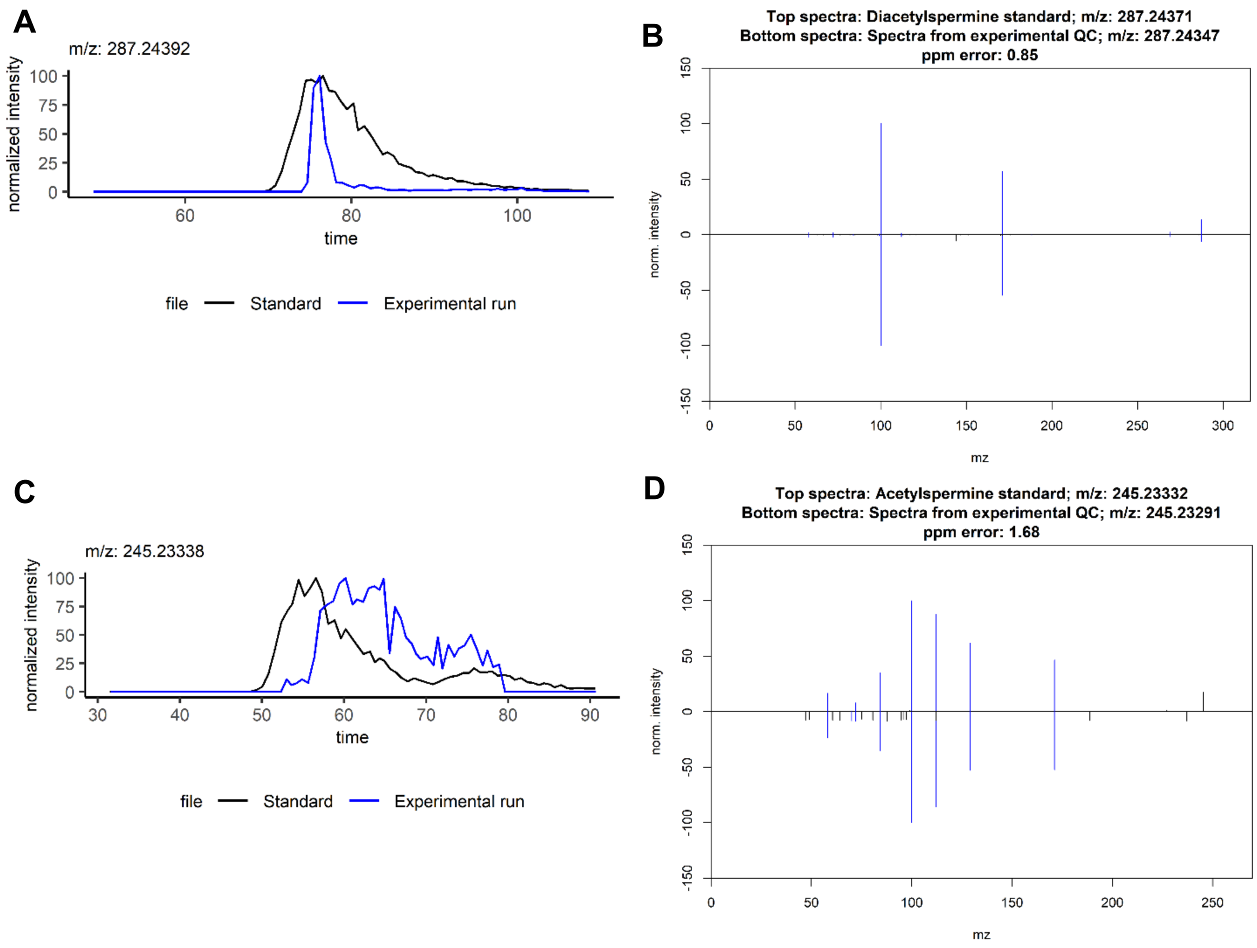

Figure S1: Mass spectrometric identification of diacetylspermine and acetylspermine

(A) MS1 trace of diacetylspermine standard (blue) and pooled experimental QC sample (black) for 287.24392+/- 5ppm *m/z*. (B) MS2 spectra of diacetylspermine standard (top) and spectra from pooled experimental QC sample (bottom). Fragment matches within 6ppm are marked in blue. (C) MS1 trace of acetylspermine standard (blue) and pooled experimental QC sample (black) for 245.23338+/- 5ppm *m/z*. Peak shifting is within normal variation for our chromatography at the beginning of the gradient. (D) MS2 spectra of acetylspermine standard (top) and spectra from pooled experimental QC sample (bottom). Fragment matches within 6ppm are marked in blue.

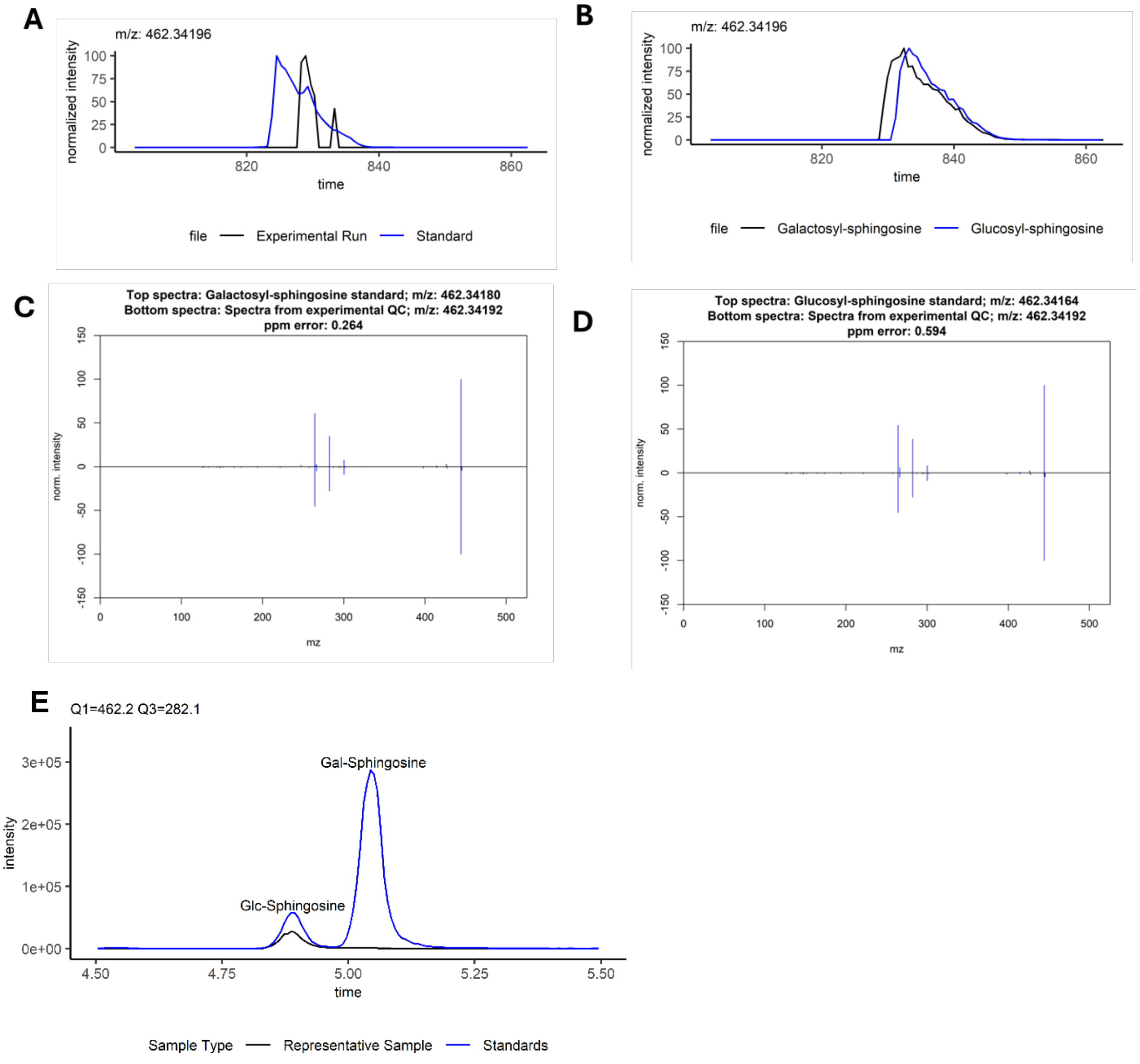

Figure S2: Mass spectrometric identification of glucosyl-sphingosine

(A) MS1 trace of galactosyl-sphingosine standard (blue) and concurrently run pooled experimental QC sample (black) for 462.34196+/- 5ppm *m/z*. (B) MS1 trace of galactosyl-sphingosine standard (black) and glucosyl-sphingosine standard (blue) for 462.34196 +/- 5ppm *m/z*. (C) MS2 spectra of galactosyl-sphingosine standard (top) and spectra from pooled experimental QC sample (bottom). Fragment matches within 6ppm are marked in blue. (D) MS2 spectra of glucosyl-sphingosine standard (top) and spectra from pooled experimental QC sample (bottom). Fragment matches within 6ppm are marked in blue. (E) Resolution of glucosyl- and galactosyl-sphingosine using a HALO HILIC column (see next page for methods). Pooled standards of glucosyl-sphingosine and galactosyl-spingosine are shown in blue, with a representative sample shown in black. Retention times indicate clinical samples contain glucosyl-sphingosine.

Supplemental methods (Resolution of glucosyl vs galactosyl sphingosine)

Glucosyl-sphingosine and galactosyl sphingosine were purchased from Avanti Polar Lipids Inc (Alabaster AB). 20ng of glucosyl-sphingosine and 100ng of galactosyl-sphingosine were dissolved in 500 µL of 80% methanol. 1ul of the standard as well as high-intensity clinical samples were injected onto the HPLC-MS/MS system to differentiate glucosyl- and galactosyl-sphingosine. A HALO HILIC column (Advanced Material Technology Inc, Wilmington, DE) (2.7 µM, 4.6 x 100 mm) was connected to Shimadzu HPLC pump (20 ADXR) with the autosampler (20 ACXR) interfaced with an AP 4000Qtrap mass spectrometer. HPLC solvent gradient was controlled from 95% B : 0.1% formic acid and 1mM ammonium formate in 95% acetonitrile 5% water to 85% in 6 min. The solvent A was 0.1% formic acid and 1mM ammonium formate in water. The solvent flow rate was 1mL/min. Mass spectral detection was carried out positive ion MRM mode (462.2/282.1) with DP (V) 76, CE(V) 31.

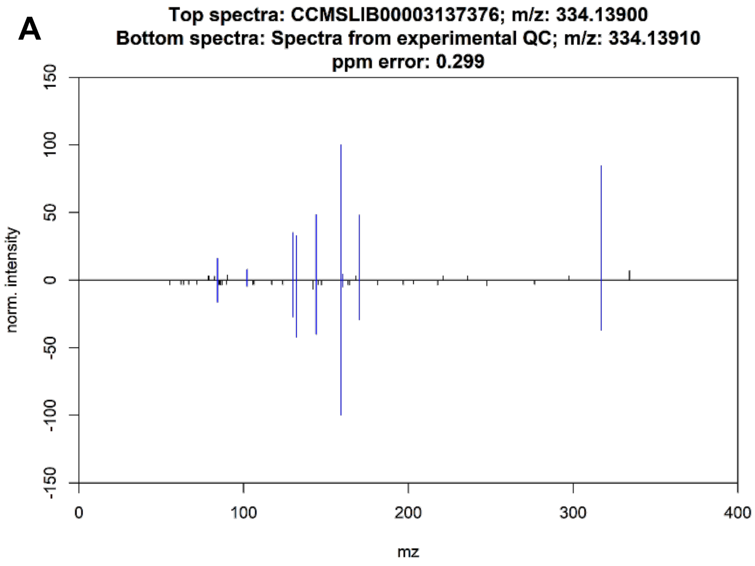

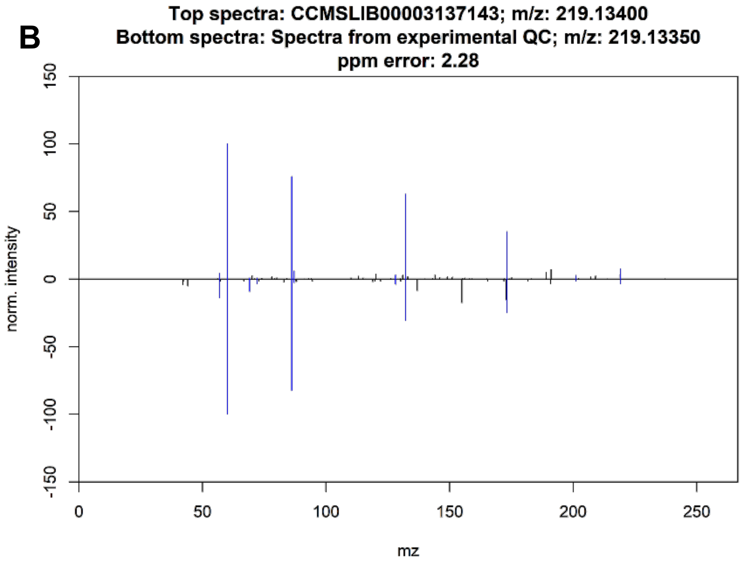

Figure S3: Mass spectrometric library matches of putative dipeptide features **[continued on next page]**

C

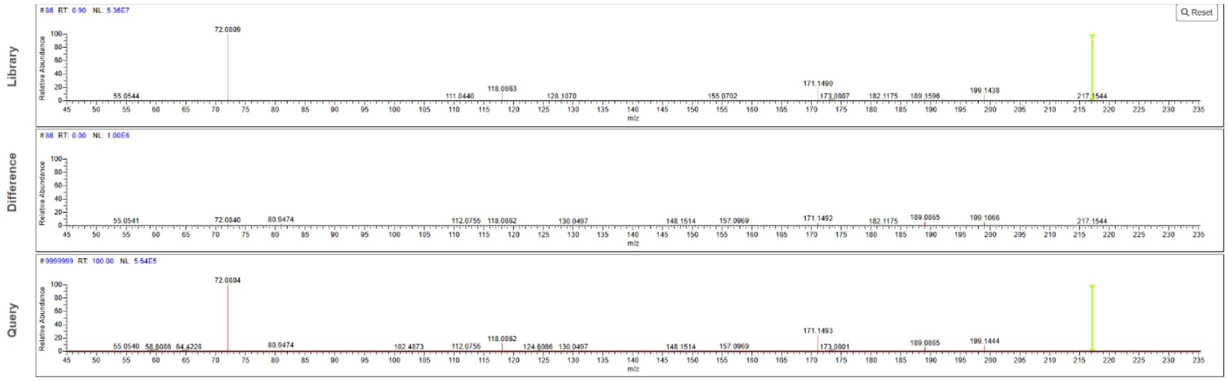

Figure S3: Mass spectrometric library matches of putative dipeptide features

(A) MS2 butterfly plot of GNPS library spectra CCMSLIB00003137376 “Spectral Match to Trp-Glu from NIST14” (top) to experimental spectra of putative Trp-Glu (bottom) (B) MS2 butterfly plot of GNPS library spectra CCMSLIB00003137143 “Spectral Match to Ser-Leu from NIST14” (top) to experimental spectra of putative Ser-Leu (bottom). Note that leucine and isoleucine are difficult to differentiate through MS2 fragment matching and thus this dipeptide may also be Ser-Ile (C) Thermo mzCloud library match for a MS2 spectra of putative Val-Val with a reference spectra of Val-Val. For A and B, blue lines indicate matches of MS2 fragments within a mass tolerance of 6ppm.

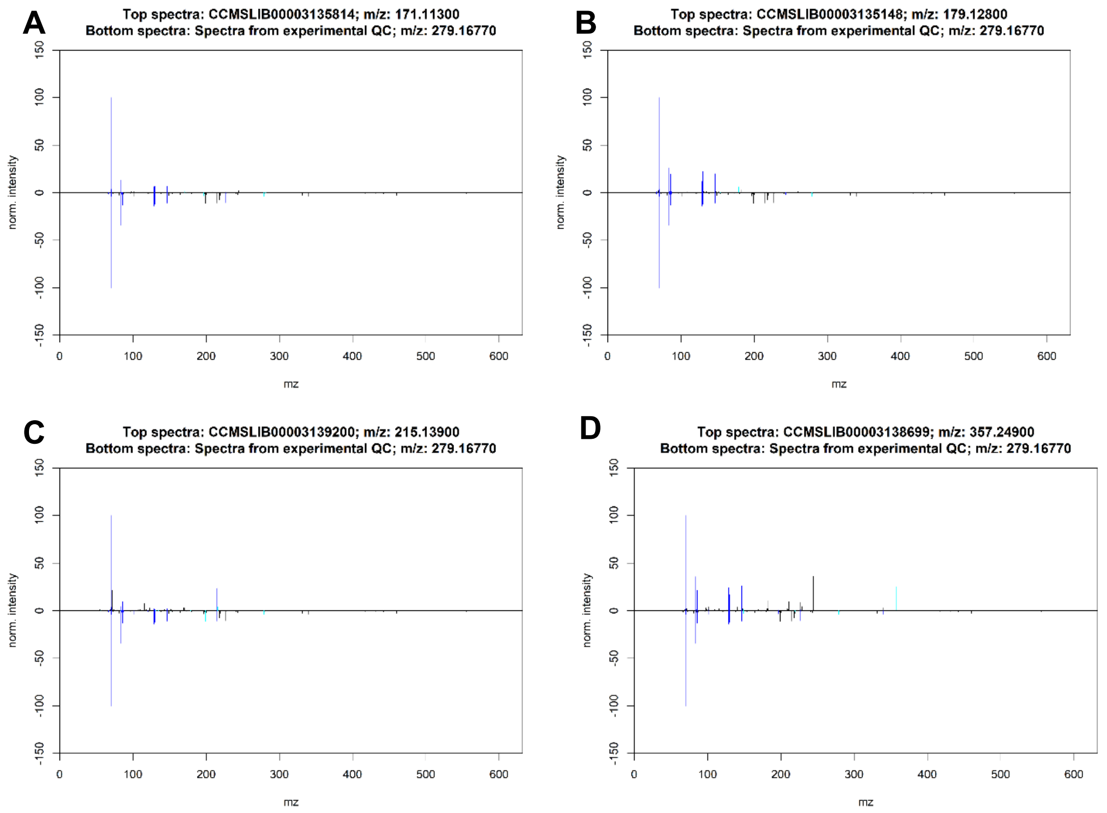

Figure S4: Mass spectrometric library matches of putative peptide-like features **[continued on next page]**

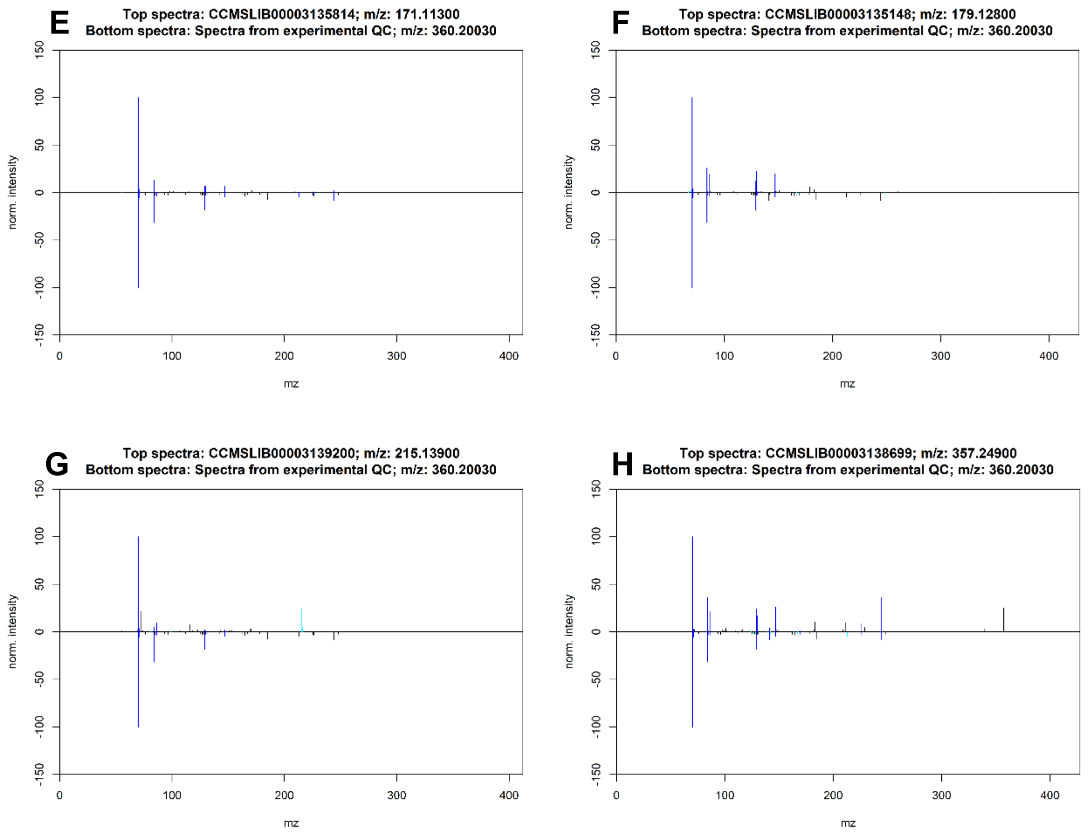

Figure S4: Mass spectrometric library matches of putative peptide-like features

Butterfly plot of four peptide-like GNPS library spectra (CCMSLIB00003135814 “Spectral Match to Pro-Pro-Lys from NIST14”; CCMSLIB00003135148 “Spectral Match to Pro-Ile-Lys from NIST14”; CCMSLIB00003139200 “Spectral Match to Pro-Val from NIST14”; CCMSLIB00003138699 “Spectral Match to Ile-Pro-Lys from NIST14”) with a spectra obtained from a QC sample created from experimental sample for the “peptide-like 556.3 Da” molecule (A-D) and “peptide-like 1077.6 Da” (E-H). Blue lines indicate matches of MS2 fragments within a mass tolerance of 0.02 Da (note for library spectra, a flat mass tolerance is used instead of ppm error due to potential differences in instrumentation and calibration). Cyan lines indicate masses that differ from a matching fragment by the *m/z* difference between the two molecules as utilized by the GNPS modified cosine score. Due to the close retention time and high similarity of these two features, they may be part of a source decay family for a larger peptide that fragments within the ion source of our instrumentation.

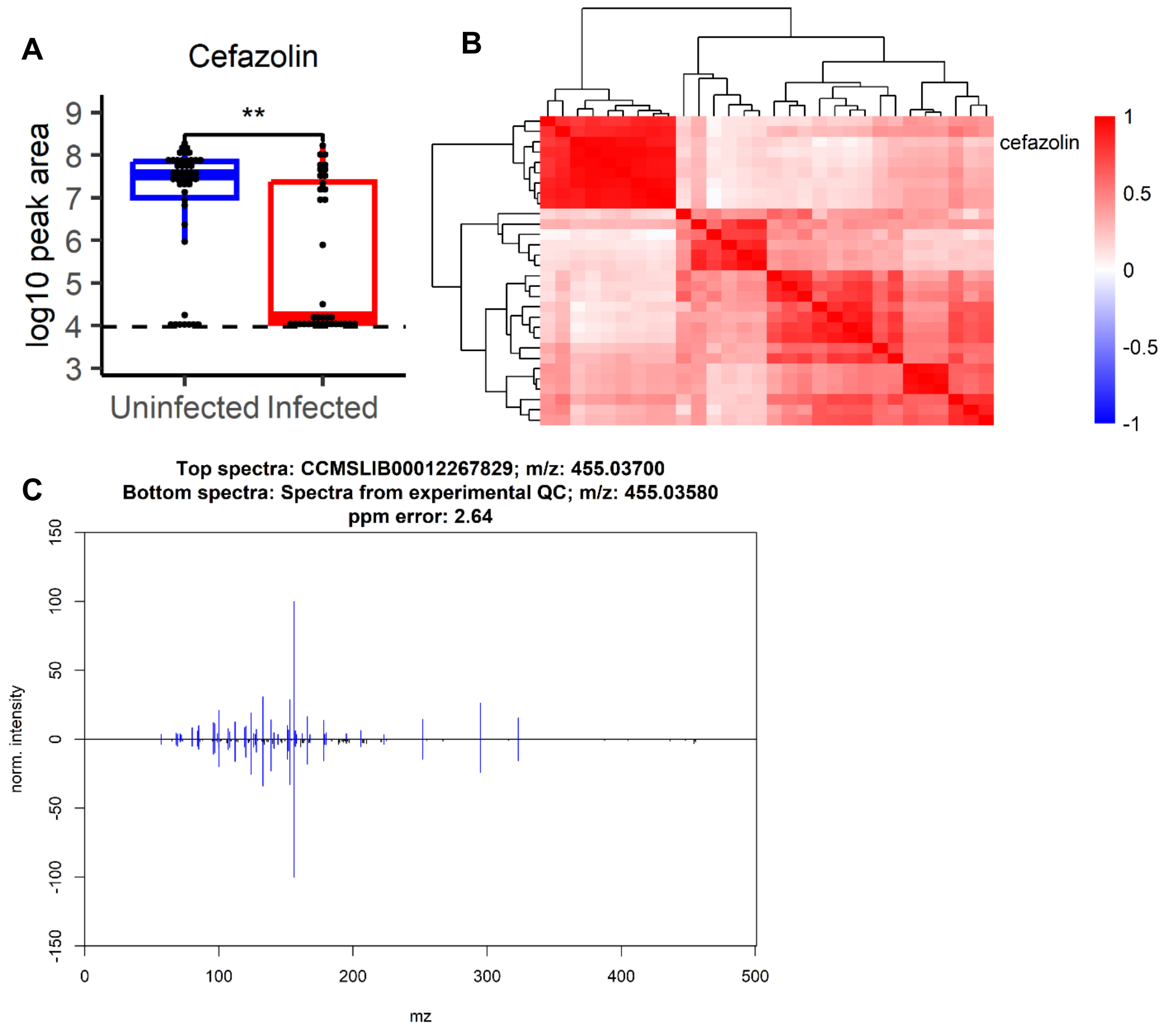

Figure S5: Identification of cefazolin and associated features in seroma fluid samples collected at time of implant removal

(A) Dot plot and box and whisker plot showing prevalence of cefazolin in samples at time of implant removal. Significance is denoted by FDR (BH method) adjusted Wilcoxon Rank Sum P values (** $P < 0.01$). Box plots exclude outliers. Dashed lines indicate mean peak area in five aligned methanol blanks. (B) Correlation between cefazolin and other features that are decreased 4-fold with FDR adjusted $P < 0.01$ in infected samples compared to uninfected samples. Eight additional features cluster with cefazolin, which may constitute impurities or products of degradation or metabolism (Table S2) (C) MS2 butterfly plot of GNPS library spectra CCMSLIB00012267829 “cefazolin [M+H]+” to experimental spectra of putative cefazolin. Blue lines indicate matches of MS2 fragments within a mass tolerance of 6ppm.

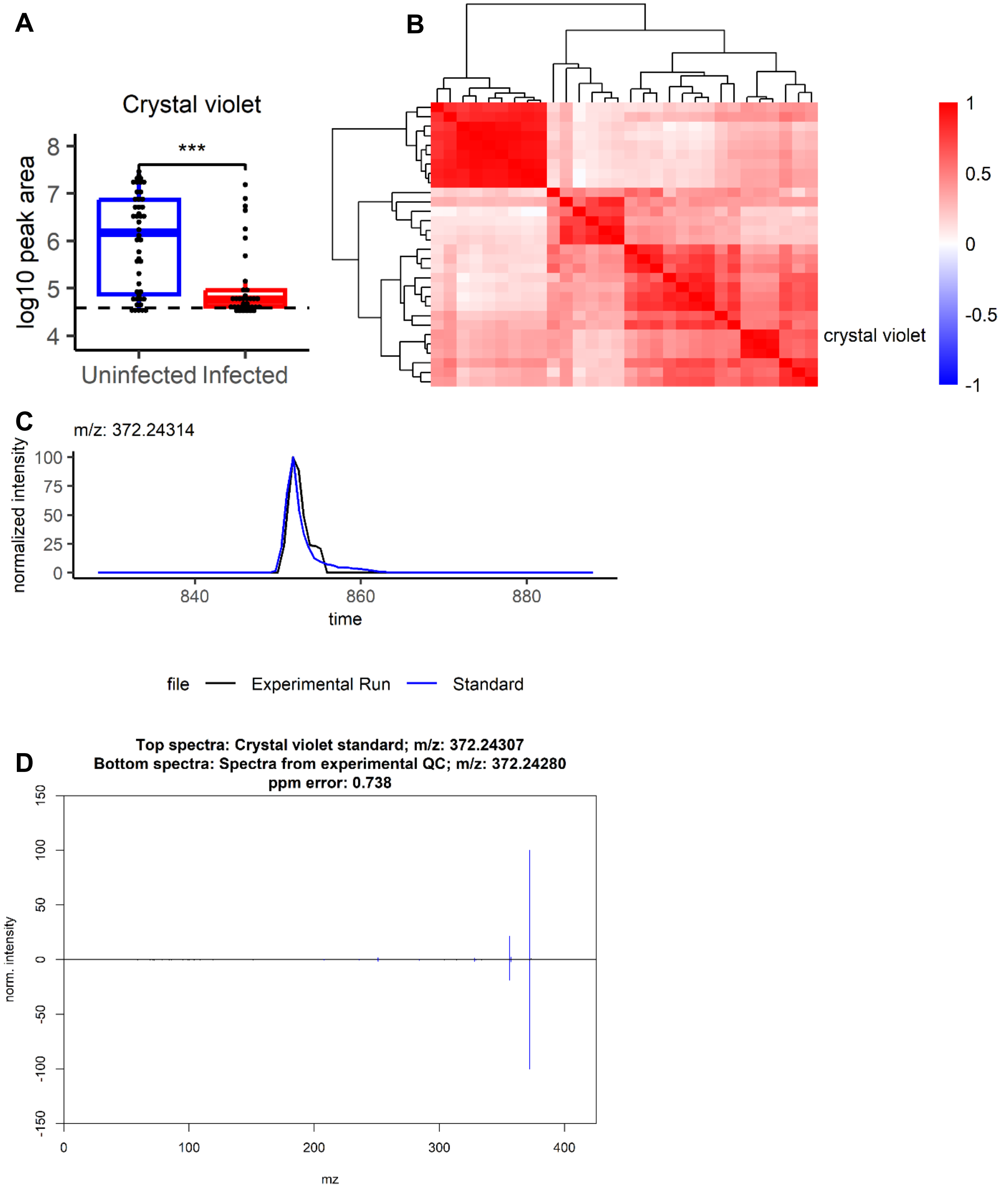

Figure S6: Identification of crystal violet and associated features in seroma fluid samples collected at time of implant removal **[continued on next page]**

E

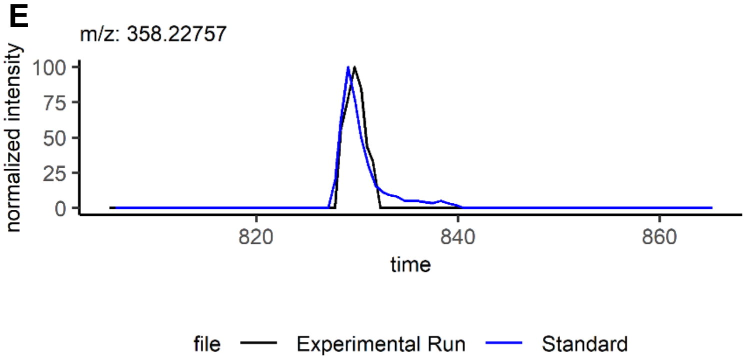

F

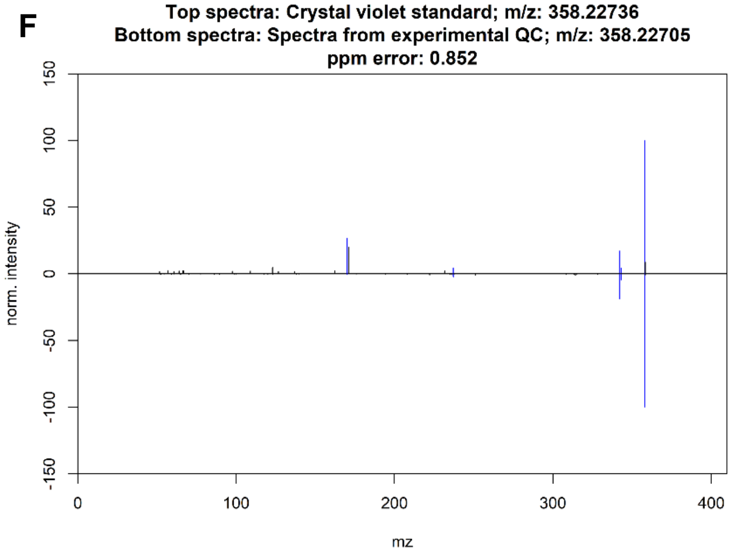

Figure S6: Identification of crystal violet and associated features in seroma fluid samples collected at time of implant removal

(A) Dot plot and box and whisker plot showing prevalence of crystal violet in samples at time of implant removal. Significance is denoted by FDR (BH method) adjusted Wilcoxon Rank Sum P values (*** P < 0.001). Box plots exclude outliers. Dashed lines indicate mean peak area in five aligned methanol blanks. (B) Correlation between crystal violet and other features decreased 4-fold with FDR adjusted P < 0.01 in infected samples compared to uninfected samples. Two additional features cluster crystal violet, which have m/z values consistent with demethylated versions of crystal violet (Table S3). (C) MS1 trace of crystal violet standard (blue) and pooled experimental QC sample (black) for 372.24314+/- 5ppm *m/z*. (D) MS2 spectra of crystal violet standard (top) and spectra from pooled experimental QC sample (bottom). Fragment matches within 6ppm are marked in blue. (E) MS1 trace of crystal violet standard (blue) and pooled experimental QC sample (black) for 358.22757 +/- 5ppm *m/z*. This peak is putative desmethyl-crystal violet and is present in commercially available crystal violet (F) MS2 spectra for putative desmethyl-crystal violet present in a crystal violet standard (possibly an impurity in commercially available crystal violet) (top) and spectra from pooled experimental QC sample (bottom). Fragment matches within 6ppm are marked in blue.

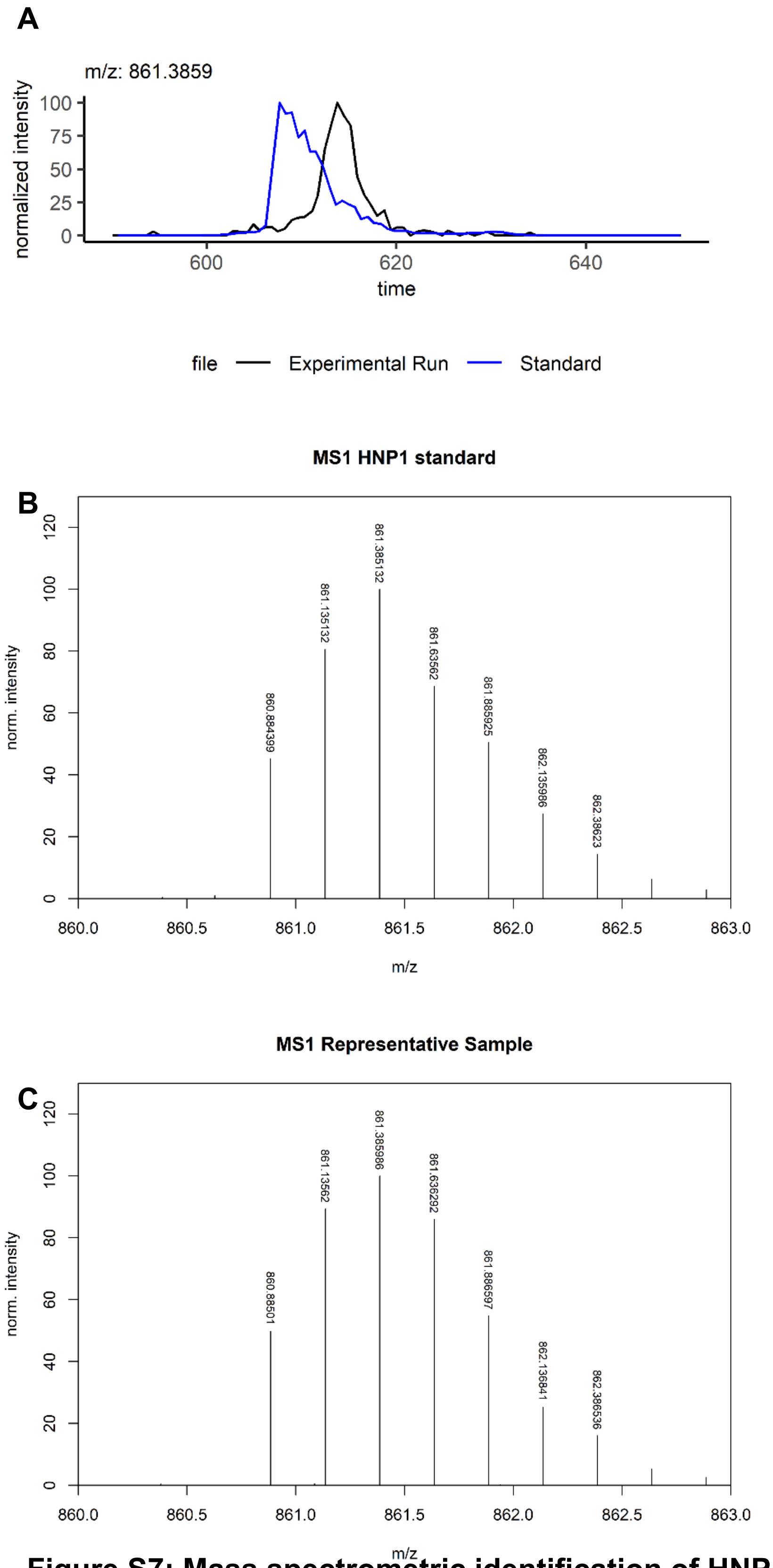

Figure S7: Mass spectrometric identification of HNP1-3 [continued on next page]

D

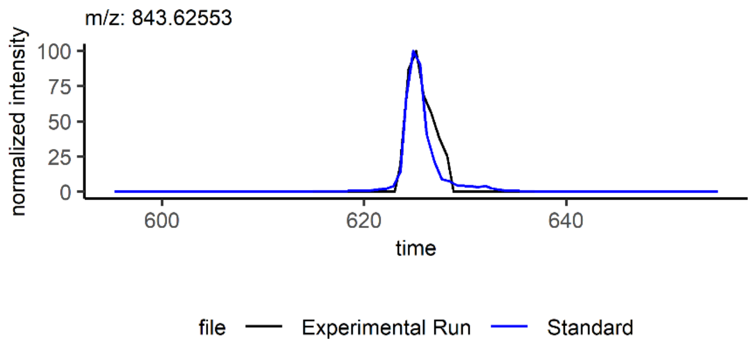

E

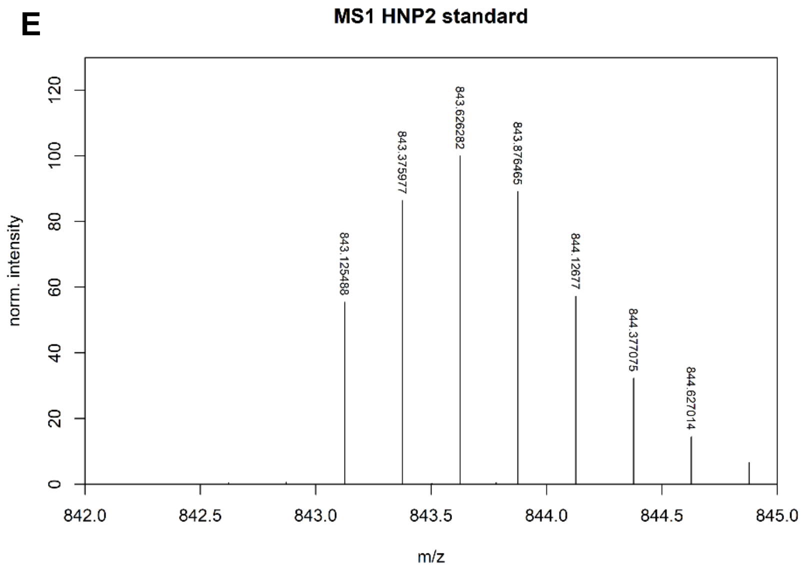

F

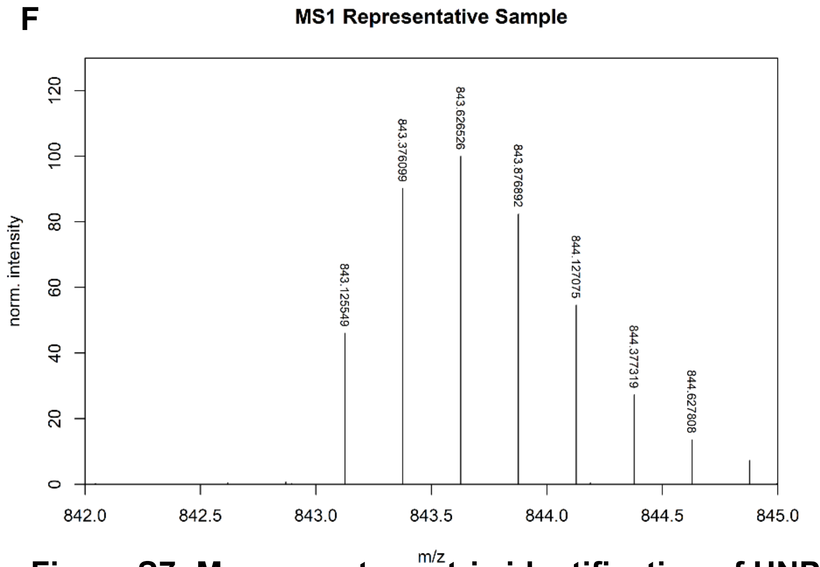

Figure S7: Mass spectrometric identification of HNP1-3 [continued on next page]

G

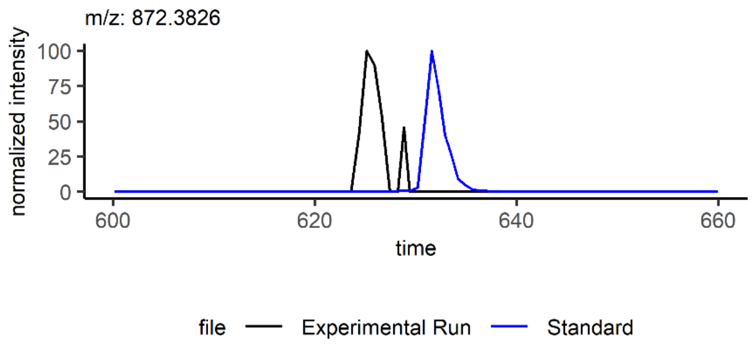

H

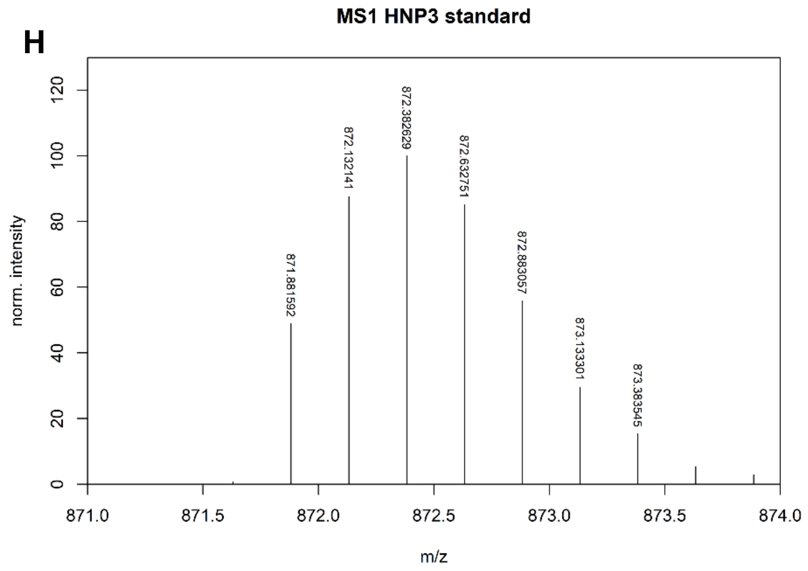

I

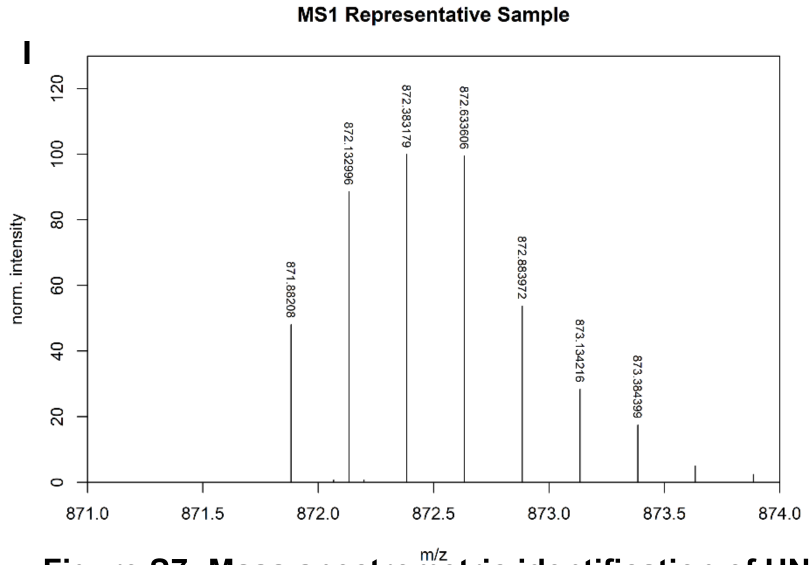

Figure S7: Mass spectrometric identification of HNP1-3 [continued on next page]

Figure S7: Mass spectrometric identification of HNP1-3

(A) MS1 trace of HNP1 standard (blue) and pooled experimental QC sample (black) for 861.3859+/- 5ppm *m/z*. (B) MS1 isotope abundance for HNP1 standard. These isotopologues are consistent with 4+ charged HNP1. (C) MS1 isotope abundance for experimental HNP1 from the highest abundance sample. These isotopologues are consistent with a 4+ charged HNP1 and match those of the HNP1 standard. (D) MS1 trace of HNP2 standard (blue) and pooled experimental QC sample (black) for 843.62553+/- 5ppm *m/z*. (E) MS1 isotope abundance for HNP2 standard. These isotopologues are consistent with 4+ charged HNP1. (F) MS1 isotope abundance for experimental HNP2 from the highest abundance sample. These isotopologues are consistent with a 4+ charged HNP2 and match those of the HNP2 standard. (G) MS1 trace of HNP3 standard (blue) and pooled experimental QC sample (black) for 872.3826+/- 5ppm *m/z*. Peak shifting is within normal range for our chromatography. (H) MS1 isotope abundance for HNP3 standard. These isotopologues are consistent with 4+ charged HNP3. (I) MS1 isotope abundance for experimental HNP3 from the highest abundance sample. These isotopologues are consistent with a 4+ charged HNP3 and match those of the HNP3 standard.

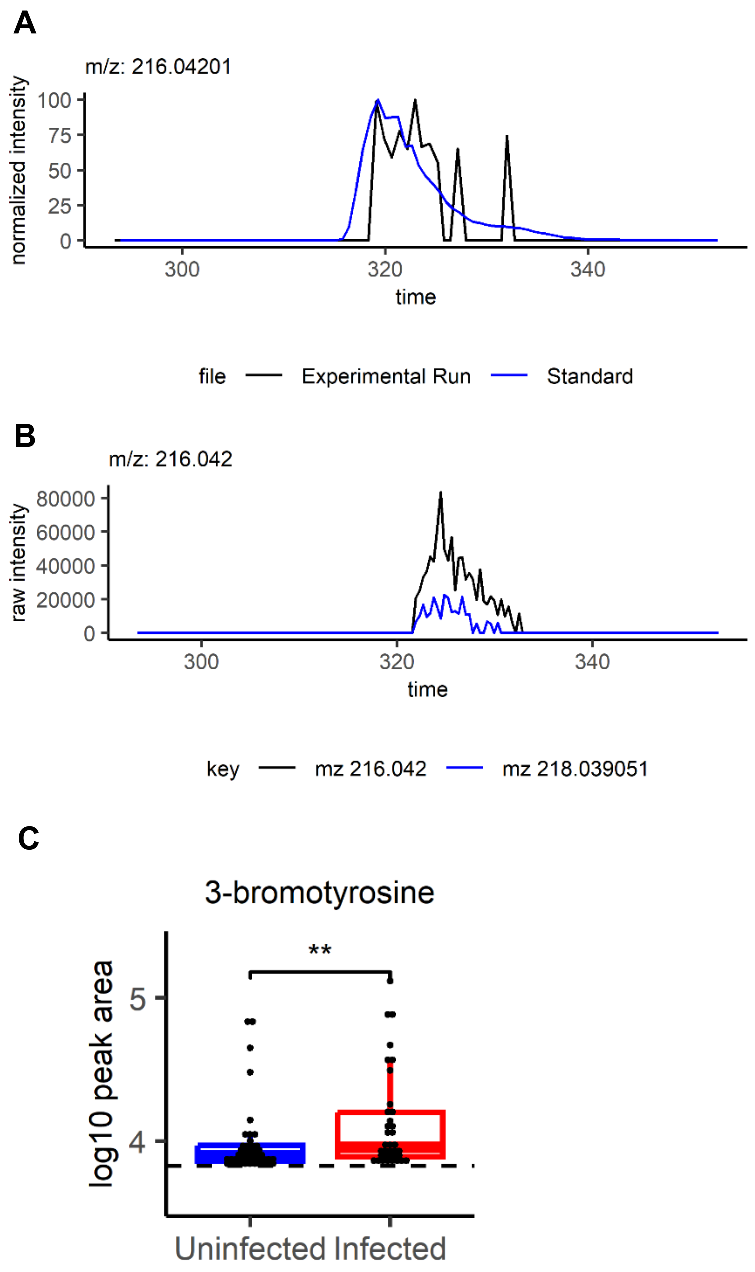

Figure S8: Mass spectrometric identification of 3-chlorotyrosine and 3-bromotyrosine [continued on next page]

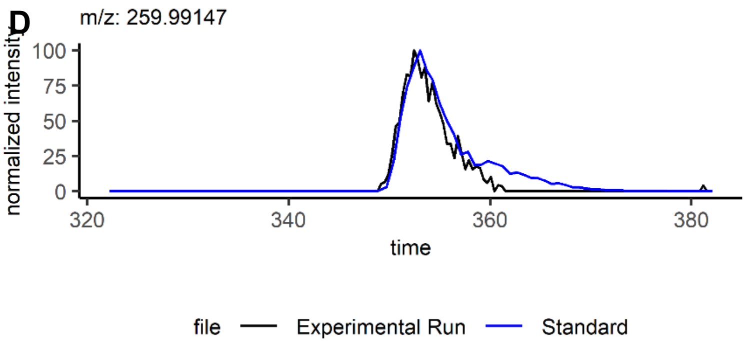

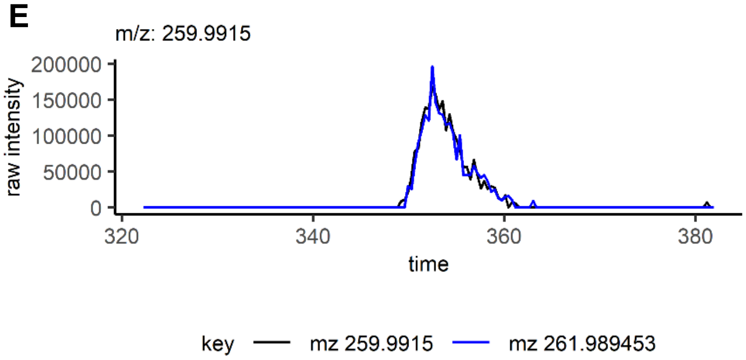

Figure S8: Mass spectrometric identification of 3-chlorotyrosine and 3-bromotyrosine

(A) MS1 trace of 3-chlorotyrosine standard (blue) and pooled experimental QC sample (black) for 216.04201+/- 5ppm *m/z*. (B) MS1 trace for *m/z* values corresponding to ^{35}Cl and ^{37}Cl isotopes showing expected ratio of abundance for 3-chlorotyrosine from the highest abundance patient sample. (C) Dot plot and box and whisker plot showing prevalence of 3-bromotyrosine. Significance for 3-bromotyrosine is denoted by unadjusted Wilcoxon Rank Sum P values (** P < 0.01). Box plots exclude outliers. Dashed lines indicate mean peak area in five aligned methanol blanks. As gap filling takes into account sample-specific background, some specimens may have calculated peak areas under this level. (D) MS1 trace of 3-bromotyrosine standard (blue) and highest abundance patient sample for 259.99147+/- 5ppm *m/z*. (E) MS1 trace for m/z values corresponding to ^{79}Br and ^{81}Br isotopes showing expected ratio of abundance for 3-bromotyrosine from the highest abundance patient sample

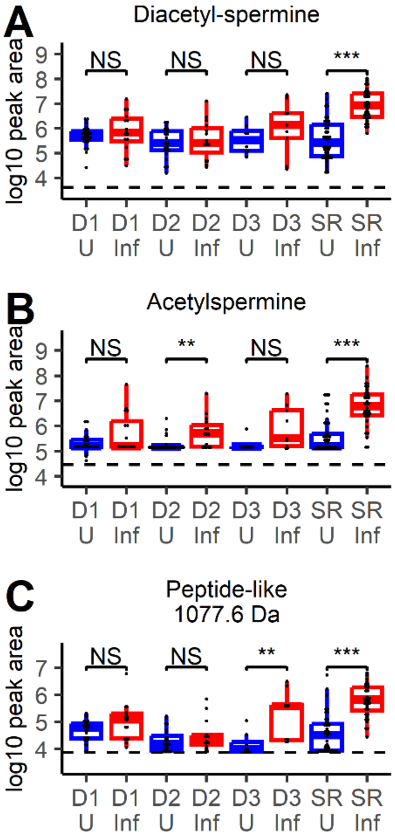

Figure S9: Biomarkers of infection that are not consistently increased in longitudinal drain fluid samples collected prior to implant removal in patients progressing to infection

Dot plot and box and whisker plot showing prevalence of (A) diacetylspermine, (B) acetylspermine, and (C) Peptide-like 1077.6 Da biomarkers from longitudinally collected drain fluid in cohort2 (“D1” drain collection 1, “D2” drain collection 2, “D3” drain collection 3) and seroma fluid collected from breasts at the time of implant removal (“SR”) in breasts from both cohort1 and cohort2. Specimens are additionally stratified on whether they were from breasts with implants removed due to infection (“Inf”, red) or remaining uninfected (“U”, blue). Significance is denoted by FDR (BH method) adjusted Wilcoxon Rank Sum P values (** P < 0.01, *** P < 0.001). Exact FDR adjusted P values may be found in Table 5. Box plots exclude outliers. Dashed lines indicate mean peak area in five aligned methanol blanks. As gapfilling takes into account sample-specific background, some specimens may have calculated peak areas under this level.

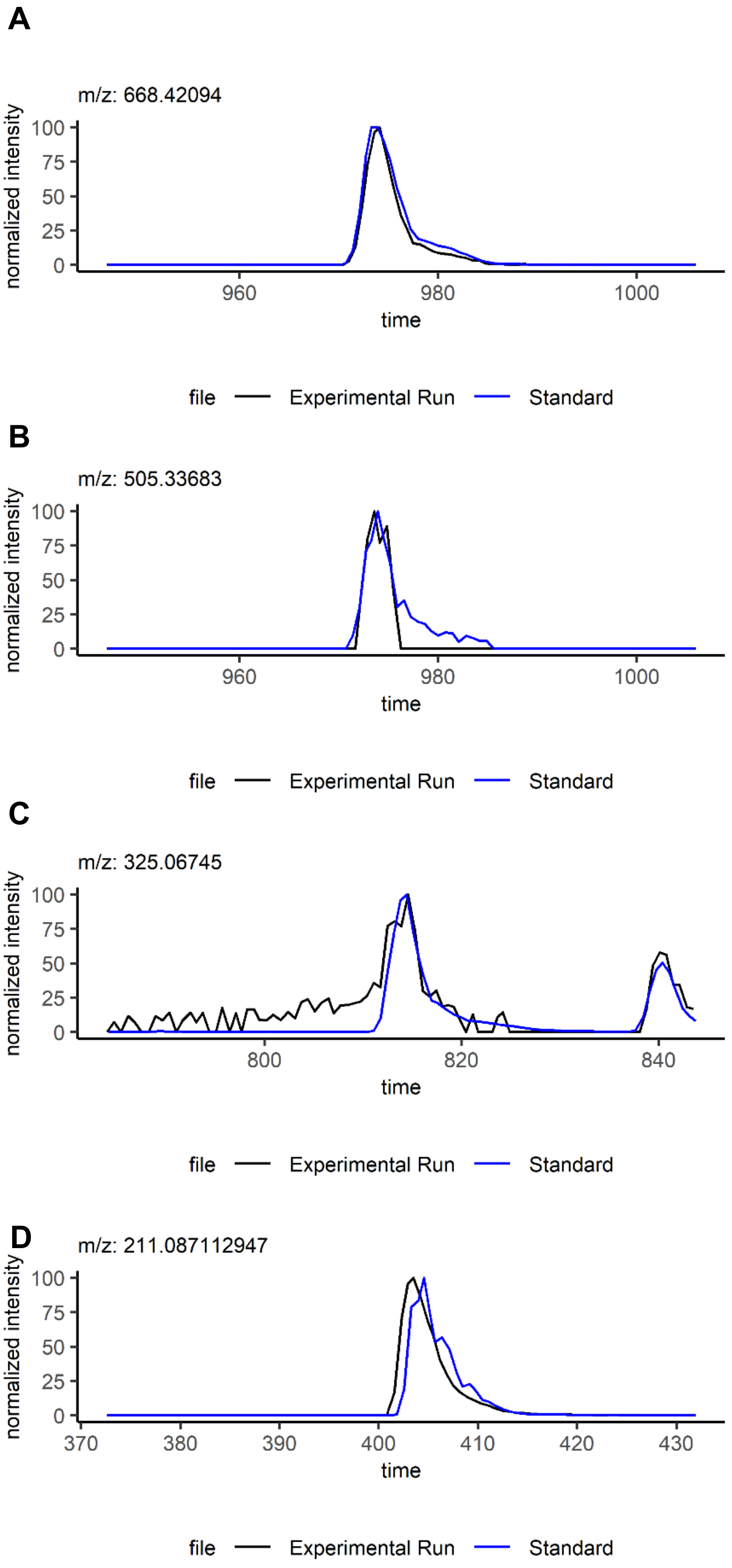

Figure S10: Mass spectrometric identification of *Pseudomonas aeruginosa* metabolites [continued on next page]

E

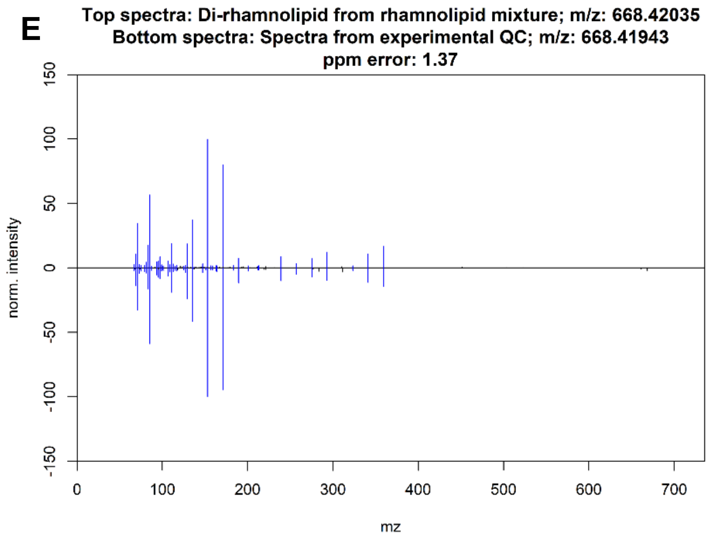

F

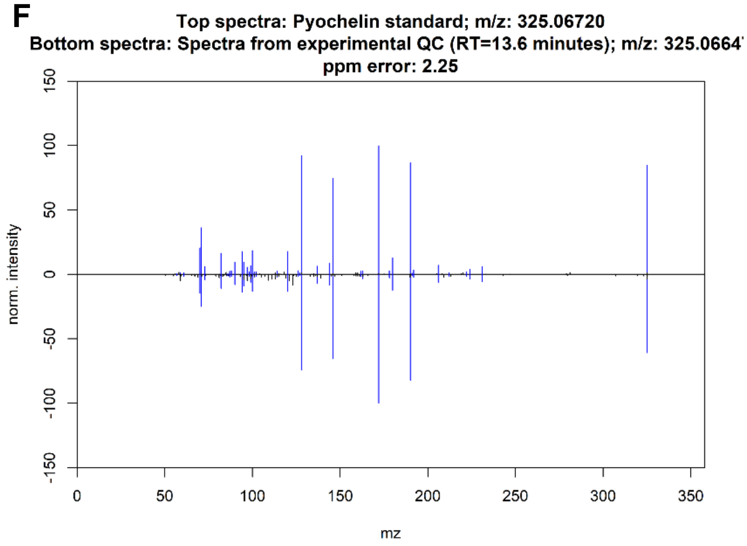

G

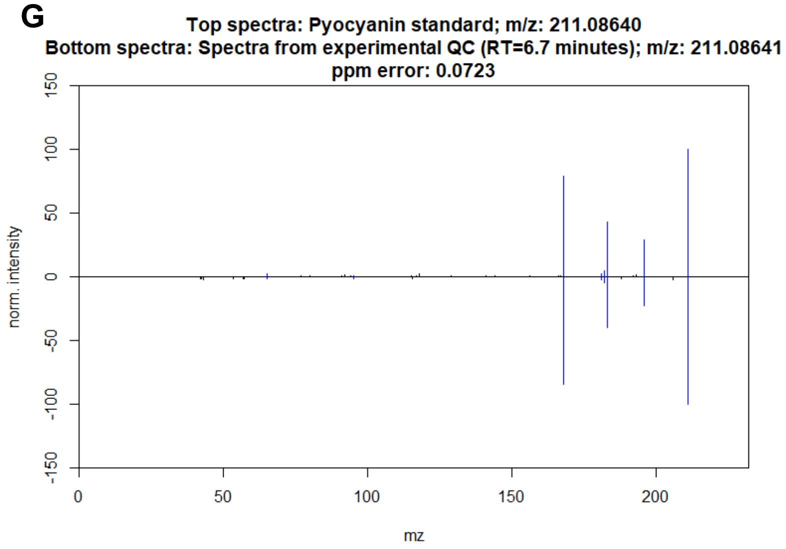

Figure S10: Mass spectrometric identification of *Pseudomonas aeruginosa* metabolites [continued on next page]

Figure S10: Mass spectrometric identification of *Pseudomonas aeruginosa* metabolites

(A) MS1 trace of rhamnolipid mixture standard (blue) and pooled experimental QC sample (black) for 668.42094+/- 5ppm *m/z*. This corresponds to the [M+NH4]+ adduct of di-rhamnolipid (the highest intensity adduct as selected by Compound Discoverer) (B) MS1 trace of rhamnolipid mixture standard (blue) and pooled experimental QC sample (black) for 505.33683+/- 5ppm *m/z*. (C) MS1 trace of pyochelin standard (blue) and pooled experimental QC sample (black) for 325.06745+/- 5ppm *m/z*. Pyochelin has multiple known isoforms (appearing as a double peak at ~13.6 and ~14.0 minutes); for our study we integrated the highest intensity isoform (RT: 13.6 minutes). (D) MS1 trace of pyocyanin standard (blue) and pooled experimental QC sample (black) for 211.08711+/- 5ppm *m/z*. (E) Butterfly plot of MS2 spectra for di-rhamnolipid from a rhamnolipid mixture standard (top) and a spectra from pooled experimental QC sample (bottom). Fragment matches within 6ppm are marked in blue. (F) Butterfly plot of MS2 spectra for pyochelin from a pyochelin standard (top) and spectra from pooled experimental QC sample (bottom). Fragment matches within 6ppm are marked in blue. (G) Butterfly plot of MS2 spectra for pyocyanin from a pyocyanin standard (top) and spectra from pooled experimental QC sample (bottom). Fragment matches within 6ppm are marked in blue.

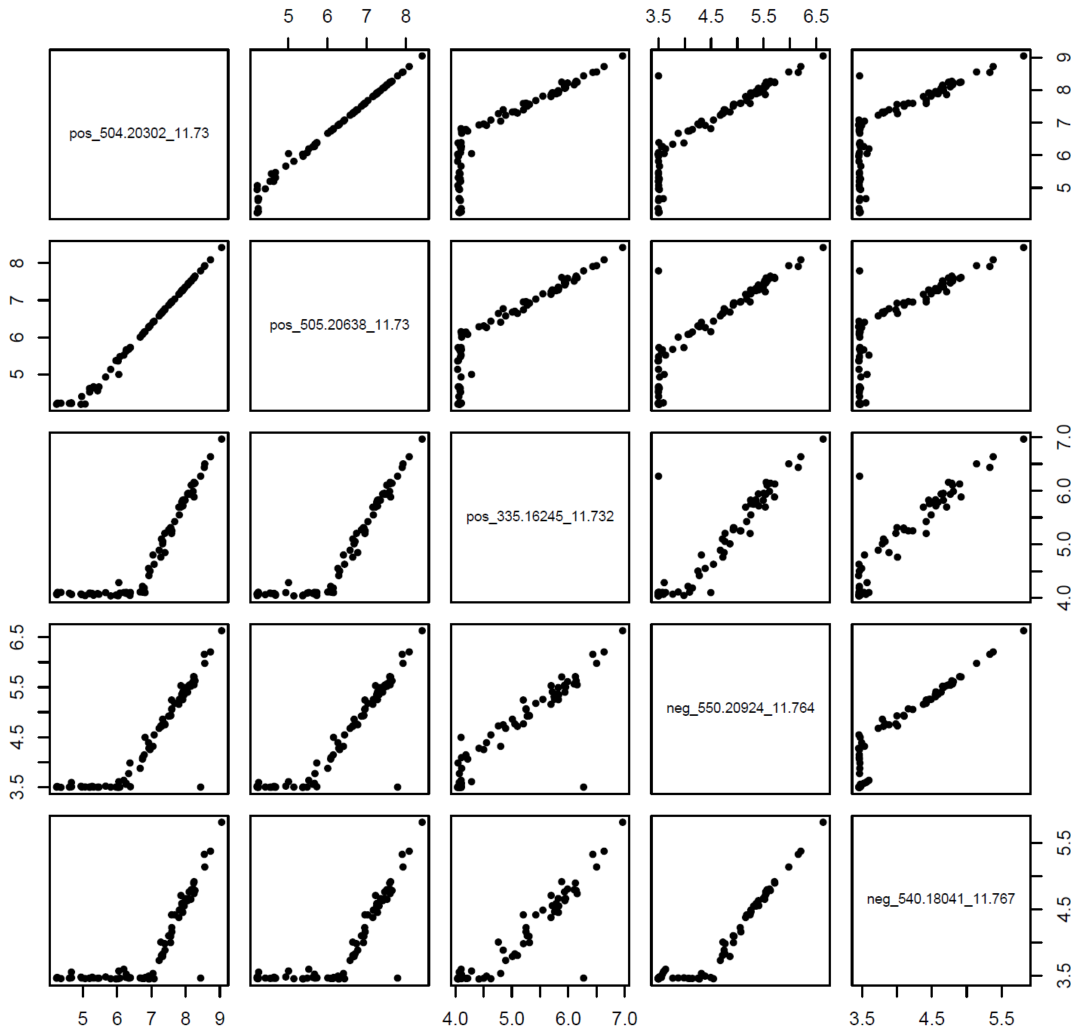

Figure S11: Example of a single putative molecule giving rise to multiple features

These graphs show high intra-specimen correlation between a feature (pos_504.20302_11.73, calculated molecular weight of 504 Da) with several other features called by Compound Discoverer that were not associated with the initial molecule and represent partial duplications within the data that may have an unknown effect on statistical analysis. In this case, the 13C isotope, two negative mode adducts that are most likely [M+FA-H]- and [M+Cl]-, and a putative in-source decay fragment (pos_335.16245_11.732, calculated molecular weight of 335 Da) were not associated with the primary feature. Highly correlated families such as these were collapsed into the most intense feature to avoid partial duplications of data among features.

TABLE S1: Microbiology of seroma fluid specimens collected at the time of breast implant removal separated by mono- and poly-microbial culture

Bacterial Growth[A]	Infected n=36 (%)			Uninfected (n=46) Planned removal at second-stage reconstruction n=34 (%)			Uninfected (n=46) Non-infectious complications n=10 (%)			Uninfected (n=46) Elective removal contralateral to infection n=2 (%)		
No growth	4 (11.1)			20 (58.8)			5 (50)			1 (50)		
Monomicrobial	20 (55.6)			9 (26.5)			1 (10)			1 (50)		
Polymicrobial	12 (33.3)			5 (14.7)			4 (40)			0 (0)		
**Bacterial identity[B]**												
Staphylococcus	Total	M	P	Total	M	P	Total	M	P	Total	M	P
Coagulase-negative staphylococcus	10 (27.8)	4	6	6 (17.6)	3	3	3 (30)	-	3	-	-	-
Staphylococcus aureus	4 (11.1)	3	1	-	-	-	-	-	-	-	-	-
Staphylococcus lugdunensis	2 (5.6)	-	2	-	-	-	1 (10)	-	1	-	-	-
Pseudomonas aeruginosa	8 (22.2)	5	3	-	-	-	1 (10)	-	1	-	-	-
Corynebacterium spp.	5 (13.9)	3	2	-	-	-	2 (20)	-	2	-	-	-
Cutibacterium spp.	5 (13.9)	3	2	9 (26.5)	5	4	2 (20)	1	1	1 (50)	1	-
Enterococcus faecalis	2 (5.6)	-	2	-	-	-	-	-	-	-	-	-
Proteus mirabilis	2 (5.6)	1	1	-	-	-	2 (20)	-	2	-	-	-
Streptococcus spp.	2 (5.6)	-	2	1 (2.9)	1	-	-	-	-	-	-	-
Bacillus cereus group	1 (2.8)	-	1	-	-	-	-	-	-	-	-	-
Citrobacter koseri	1 (2.8)	-	1	-	-	-	-	-	-	-	-	-
Enterobacter spp.	1 (2.8)	-	1	-	-	-	1 (10)	-	1	-	-	-
Finegoldia magna	1 (2.8)	-	1	-	-	-	-	-	-	-	-	-
Klebsiella aerogenes	1 (2.8)	1	-	-	-	-	-	-	-	-	-	-
Klebsiella pneumoniae	1 (2.8)	-	1	-	-	-	-	-	-	-	-	-
Serratia marcescens	1 (2.8)	-	1	-	-	-	-	-	-	-	-	-
Micrococcus luteus	-	-	-	1 (2.9)	-	1	-	-	-	-	-	-
Paenibacillus lautus	-	-	-	1 (2.9)	-	1	-	-	-	-	-	-
Peptoniphulus asaccharolyticus	-	-	-	-	-	-	1 (10)	-	1	-	-	-

[A]Specimens were considered positive for an organism if it grew from cultured seroma fluid or from implant swabs. [B]Percentages do not add up to 100% due to polymicrobial growth. “M” columns represent organisms isolated in monomicrobial culture. “P” columns represent organisms isolated in polymicrobial culture. Exact polymicrobial culture results are listed on the next page.

NOTE: in isolates labeled as "spp." below, MALDI-TOF was unable to give a species-level identity. This differs from Table 3 and Table S1, where "spp." is used to cluster similar organisms (i.e. *Corynebacterium* spp, *Cutibacterium* spp.) for table readability

Polymicrobial isolates (Infected):

- Bacillus cereus group, Enterobacter spp., Staphylococcus capitis
- Citrobacter koseri, Klebsiella pneumoniae
- Corynebacterium spp., Cutibacterium acnes
- Corynebacterium tuberculostearicum, Cutibacterium acnes, Staphylococcus epidermidis
- Enterococcus faecalis, Staphylococcus epidermidis, Streptococcus sanguis
- Enterococcus faecalis, Staphylococcus lugdunensis
- Finegoldia magna, Staphylococcus lugdunensis
- Proteus mirabilis, Pseudomonas aeruginosa
- Pseudomonas aeruginosa, Staphylococcus epidermidis
- Pseudomonas aeruginosa, Staphylococcus epidermidis
- Serratia marcescens, Staphylococcus aureus
- Staphylococcus epidermidis, Streptococcus agalactiae

Polymicrobial isolates (Planned removal at second stage reconstruction):

- Cutibacterium acnes, Cutibacterium avidum, Cutibacterium granulosum
- Cutibacterium acnes, Micrococcus luteus
- Cutibacterium acnes, Staphylococcus epidermidis
- Cutibacterium avidum, Staphylococcus epidermidis
- Paenibacillus lautus, Staphylococcus epidermidis

Polymicrobial isolates (Non-infectious complications):

- Corynebacterium jeikeium, Cutibacterium avidum, Enterobacter cloacae, Staphylococcus epidermidis, Staphylococcus lugdunensis
- Corynebacterium simulans, Corynebacterium xerosis, Proteus mirabilis, Staphylococcus epidermidis
- Peptoniphulus asaccharolyticus, Proteus mirabilis
- Pseudomonas aeruginosa, Staphylococcus epidermidis

TABLE S2: Features decreased in infection that are highly correlated with cefazolin

feature	m/z	Calculated Molecular weight	RT	correlation to cefazolin
cefazolin	455.0368	454.0295	9.9	1
neg_354.0741_7.61	353.0668	354.0741	7.61	0.98
pos_354.07435_7.558	355.0816	354.0744	7.558	0.97
pos_147.97643_7.014	148.9837	147.9764	7.014	0.95
neg_131.98163_7.475	130.9744	131.9816	7.475	0.94
pos_342.07442_7.314	343.0817	342.0744	7.314	0.89
pos_131.98152_7.428	132.9888	131.9815	7.428	0.88
pos_454.02961_9.462	455.0369	454.0296	9.462	0.81
pos_261.94736_7.417	262.9547	261.9474	7.417	0.77

TABLE S3: Features decreased in infection that are highly correlated with crystal violet

feature	m/z	RT	correlation to crystal violet	Notes
Crystal violet	372.2431	14.303	1	
pos_357.22029_13.924	358.2276	13.924	0.96	Mass is consistent with mono-demethylated crystal violet
pos_343.20481_13.533	344.2121	13.533	0.93	Mass is consistent with di-demethylated crystal violet

Table S4: Table of clinical events in relation to drain fluid collections in all breasts from cohort2

Drain collection #; Final infection status	Number of specimens[A]	Culture positive drains	Days post mastectomy (median [range])	Days prior to implant removal (median [range])	Days prior to infection diagnosis[B] (median [range])	Days prior to first erythema (median [range])	Number of breasts with eventual erythema	Days prior to first swelling (median [range])	Number of breasts with eventual swelling
Drain #1 Uninfected	28	13 (46%)	9 [6, 15]	108 [30, 461]	--	40 [21, 85]	5/28 (18%)	25 [7, 73]	5/28 (18%)
Drain #1 Infected	16	13 (81%)	11.5 [7, 15]	36 [16, 89]	35 [8, 89]	28 [-4, 85]	9/16 (56%)	26 [1, 84]	11/16 (69%)
Drain #2 Uninfected	24	18 (75%)	20 [15, 29]	86 [22, 447]	--	28.5 [14, 81]	4/24 (17%)	14 [11, 26]	3/24 (13%)
Drain #2 Infected	15	15 (100%)	18 [15, 34]	29 [7, 85]	23 [1, 85]	18 [-11, 81]	9/15 (60%)	18 [-11, 80]	11/15 (73%)
Drain #3 Uninfected	8	8 (100%)	25.5 [22, 55]	89 [15, 433]	--	9.5 [-8, 72]	4/8 (50%)	-8 [-28, 12]	2/8 (25%)
Drain #3 Infected	9	9 (100%)	28 [22, 52]	13 [1, 76]	12 [-6, 76]	7 [-11, 72]	5/9 (56%)	3.5 [-22, 71]	6/9 (67%)

[A]All patients have an initial drain fluid collection but drain removal results in drop-outs for further timepoints. [B]Diagnosis of infection occurred at time of implant removal in 7/16 infected patients in cohort2

Table S5: Microbiology of drain fluid collections #1-3 in cohort2

	Drains from breasts progressing to infection			Drains from breasts remaining uninfected		
**Bacterial Growth**	**Drain #1 (n=16)**	**Drain #2 (n=15)**	**Drain #3 (n=9)**	**Drain #1 (n=28)**	**Drain #2 (n=24)**	**Drain #3 (n=8)**
No growth	3 (18.8%)	0 (0%)	0 (0%)	15 (53.6%)	6 (25%)	0 (0%)
Monomicrobial	8 (50%)	7 (46.7%)	3 (33.3%)	4 (14.3%)	3 (12.5%)	1 (12.5%)
Polymicrobial	5 (31.2%)	8 (53.3%)	6 (66.7%)	9 (32.1%)	15 (62.5%)	7 (87.5%)
**Bacterial Identity**[A]						
Pseudomonas aeruginosa	5 (31.2%)	7 (46.7%)	5 (55.6%)	0 (0%)	1 (4.2%)	0 (0%)
Coagulase-neg. *Staphylococcus*	5 (31.2%)	3 (20%)	2 (22.2%)	10 (35.7%)	12 (50%)	4 (50%)
Enterococcus faecalis	4 (25%)	4 (26.7%)	3 (33.3%)	3 (10.7%)	8 (33.3%)	1 (12.5%)
Corynebacterium spp.	3 (18.8%)	3 (20%)	3 (33.3%)	0 (0%)	0 (0%)	3 (37.5%)
Staphylococcus aureus	1 (6.2%)	3 (20%)	3 (33.3%)	0 (0%)	1 (4.2%)	3 (37.5%)
Streptococcus spp.	1 (6.2%)	1 (6.7%)	0 (0%)	1 (3.6%)	0 (0%)	2 (25%)
Cutibacterium spp.	1 (6.2%)	0 (0%)	0 (0%)	1 (3.6%)	0 (0%)	0 (0%)
Enterobacter spp.	0 (0%)	3 (20%)	0 (0%)	1 (3.6%)	3 (12.5%)	1 (12.5%)
Serratia marcescens	0 (0%)	1 (6.7%)	1 (11.1%)	1 (3.6%)	1 (4.2%)	0 (0%)
Proteus mirabilis	0 (0%)	1 (6.7%)	1 (11.1%)	0 (0%)	1 (4.2%)	0 (0%)
Stenotrophomonas spp.	0 (0%)	1 (6.7%)	0 (0%)	0 (0%)	2 (8.3%)	0 (0%)
Finegoldia magna	0 (0%)	1 (6.7%)	0 (0%)	0 (0%)	0 (0%)	0 (0%)
Staphylococcus lugdunensis	0 (0%)	1 (6.7%)	0 (0%)	0 (0%)	0 (0%)	0 (0%)
Acinetobacter spp.	0 (0%)	0 (0%)	0 (0%)	2 (7.1%)	3 (12.5%)	1 (12.5%)
Rothia terrae	0 (0%)	0 (0%)	0 (0%)	1 (3.6%)	0 (0%)	0 (0%)
Pseudomonas putida	0 (0%)	0 (0%)	0 (0%)	0 (0%)	2 (8.3%)	1 (12.5%)
Unidentified[B]	0 (0%)	0 (0%)	0 (0%)	0 (0%)	2 (8.3%)	0 (0%)
Bacillus cereus group	0 (0%)	0 (0%)	0 (0%)	0 (0%)	1 (4.2%)	0 (0%)
Carnobacterium divergens	0 (0%)	0 (0%)	0 (0%)	0 (0%)	1 (4.2%)	0 (0%)
Delftia acidovorans	0 (0%)	0 (0%)	0 (0%)	0 (0%)	1 (4.2%)	0 (0%)
Enterococcus faecium	0 (0%)	0 (0%)	0 (0%)	0 (0%)	1 (4.2%)	0 (0%)
Sphingobacterium spiritivorum	0 (0%)	0 (0%)	0 (0%)	0 (0%)	1 (4.2%)	0 (0%)
Bacillus spp.	0 (0%)	0 (0%)	0 (0%)	0 (0%)	0 (0%)	2 (25%)
Granulicatella adiacens	0 (0%)	0 (0%)	0 (0%)	0 (0%)	0 (0%)	1 (12.5%)
Pseudomonas oryzihabitans	0 (0%)	0 (0%)	0 (0%)	0 (0%)	0 (0%)	1 (12.5%)

[A]Percentages do not add up to 100% due to polymicrobial growth. [B]Two bacteria in drains were unable to be identified by MALDI-TOF (1 no confident ID after 4 attempts, 1 anaerobe unable to resurrect from frozen stocks)

Table S6: Concordance of bacterial growth in drains with future infection with the same bacterium for the nine most prevalent bacteria in drains

Bacterium	Bacterium is present in 1+ drain collections from a breast AND Breast is infected and bacterium is isolated at time of implant removal	Bacterium is present in 1+ drain collections from a breast AND Breast remains uninfected or a different organism is isolated at time of implant removal	Bacterium is NOT present in any drain collections from a breast AND Breast is infected and bacterium is isolated at time of implant removal	Bacterium is NOT present in any drain collections from a breast AND Breast remains uninfected or a different organism is isolated at time of implant removal	Proportion of times a bacterial species was present in 1+ drain collections from a breast prior to the breast developing infection with the same organism isolated at time of implant removal	Proportion of times a bacterial species was present in 1+ drain collections from a breast that remained uninfected or had a different organism isolated at time of implant removal	P (Fisher Exact)
Pseudomonas aeruginosa	7	2	0	35	7/7	2/37	9.39E-07
Serratia marcescens	1	2	0	41	1/1	2/43	0.0682
Enterobacter spp.	1	4	0	39	1/1	4/43	0.114
Staphylococcus aureus	1	5	0	38	1/1	5/43	0.136
Enterococcus faecalis	1	14	0	29	1/1	14/43	0.341
Corynebacterium jeikeium	0	4	1	39	0/1	4/43	1
Staphylococcus warneri	0	4	0	40	0/0	4/44	1
Staphylococcus haemolyticus	0	6	0	38	0/0	6/44	1
Staphylococcus epidermidis	1	20	1	22	1/2	20/42	1

Table S7: Compounds identified by standards

Compounds identified by standards	Manufacturer	Catalog Number	Relevant Supplementary Figure	PubChem CID (compound only; CID excludes salts)
N1-Acetylspermine (hydrochloride)	Cayman Chemical	Item No. 17919	S1	916
N1,N12-Diacetylspermine (hydrochloride)	Cayman Chemical	Item No. 17918	S1	132680
Glucosyl(ß) Sphingosine (d18:1)	Avanti Lipids	860535P	S2	22833534
Galactosyl(ß) Sphingosine (d18:1)	Avanti Lipids	860537P	S2	5280458
Defensin HNP-1 (human) (trifluoroacetate salt)	Cayman Chemical	Item No. 24572	S7	16130476
Defensin HNP-2 human	Sigma Aldrich	D6790	S7	16130867
Defensin HNP-3 (human) (trifluoroacetate salt)	Cayman Chemical	Item No. 24575	S7	16130868
3-chloro-L-Tyrosine	Cayman Chemical	Item No. 35753	S8	110992
3-Bromotyrosine (trifluoroacetate salt)	Cayman Chemical	Item No. 22606	S8	148708
Nitrotyrosine	Cayman Chemical	Item No. 89540	----	235719
Monorhamnolipid; Dirhamnolipid[A]	Sigma Aldrich	R90	S10	162246; 5458394
Pyochelin I and II[B]	Santa Cruz Biotech	sc-506665	S10	5287441; 135499974
Pyocyanin	Sigma Aldrich	P0046-5MG	S10	6817
Crystal violet	Sigma Aldrich	C3886	S6	3468

[A]Mixture of rhamnolipids, including di- and mono-rhamnolipids. [B]Pyochelin exists as multiple interconverting isoforms.

Table S8: Compounds identified by comparisons to library spectra

Compounds identified by library spectra	Relevant Supplementary Figure	Comparison library spectra	PubChem CID (compound only; CID excludes salts)
Trp-Glu	S3	CCMSLIB00003137376 [GNPS]	7009663
Ser-Leu[A]	S3	CCMSLIB00003137143 [GNPS]	7015695
Val-Val	S3	"Val-Val" [mzCloud]	107475
Peptide-like 556.3 Da	S4	CCMSLIB00003135814 [GNPS]<br>CCMSLIB00003135148 [GNPS]<br>CCMSLIB00003139200 [GNPS]<br>CCMSLIB00003138699 [GNPS]	------
Peptide-like 1077.6 Da	S4	CCMSLIB00003135814 [GNPS]<br>CCMSLIB00003135148 [GNPS]<br>CCMSLIB00003139200 [GNPS]<br>CCMSLIB00003138699 [GNPS]	------
Cefazolin	S5	CCMSLIB00012267829 [GNPS]	33255

[A]Leucine and isoleucine are difficult to differentiate through MS2 fragment matching and thus this dipeptide may also be Ser-Ile
